# Turkey B Cell Transcriptome Profile During Turkey Hemorrhagic Enteritis Virus (THEV) Infection Highlights Upregulated Apoptosis and Breakdown Pathways That May Mediate Immunosuppression

**DOI:** 10.3390/v17030299

**Published:** 2025-02-21

**Authors:** Abraham Quaye, Brett E. Pickett, Joel S. Griffitts, Bradford K. Berges, Brian D. Poole

**Affiliations:** Department of Microbiology and Molecular Biology, Brigham Young University, Provo, UT 84602, USA; aquaye@byu.edu (A.Q.); joel_griffitts@byu.edu (J.S.G.); bradford_berges@byu.edu (B.K.B.)

**Keywords:** Turkey hemorrhagic enteritis virus (THEV), adenovirus, RNA sequencing, apoptosis, immunosuppression, ER stress, B cell

## Abstract

Infection with the turkey hemorrhagic enteritis virus (THEV) can cause hemorrhagic enteritis, which affects young turkeys. This disease is characterized by bloody diarrhea and immunosuppression (IMS), which is attributed to apoptosis of infected B cells. Secondary infections due to IMS exacerbate economic losses. We performed the first transcriptomic analysis of a THEV infection to elucidate the mechanisms mediating THEV-induced IMS. After infecting and sequencing mRNAs of a turkey B-cell line, trimmed reads were mapped to the host turkey genome, and gene expression was quantified with StringTie. Differential gene expression analysis was followed by functional enrichment analyses using gprofiler2 and DAVID from NCBI. RT-qPCR of select genes was performed to validate the RNA-seq data. A total of 2343 and 3295 differentially expressed genes (DEGs) were identified at 12 hpi and 24 hpi, respectively. The DEGs correlated with multiple biological processes including apoptosis, ER unfolded protein response, and cell maintenance. Multiple pro-apoptotic genes, including APAF1, BMF, BAK1, and FAS were upregulated. Genes that play a role in ER stress-induced unfolded protein response including VCP, UFD1, EDEM1, and ATF4 were also upregulated and may contribute to apoptosis. Our data suggest that several biological processes and pathways including apoptosis and ER response to stress are important aspects of the host cell response to THEV infection. It is possible that interplay between multiple processes may mediate apoptosis of infected B-cells, leading to IMS.

## 1. Introduction

Turkey hemorrhagic enteritis virus (THEV) belongs to the genus *Siadenovirus*, family *Adenoviridae*, and infects turkeys, chickens, and pheasants [1,2]. THEV is transmitted via the fecal–oral route and causes hemorrhagic enteritis (HE) in turkeys, a debilitating disease affecting predominantly 6–12-week-old turkey poults characterized by immunosuppression (IMS), lack of vitality, splenomegaly, intestinal lesions leading to bloody diarrhea, and up to 80% mortality [3,4,5,6]. The clinical disease usually persists in affected flocks for 7–10 days, causing death and economic losses. However, secondary bacterial infections may extend the duration of illness and increase mortality for an additional 2–3 weeks due to the immunosuppressive nature of the virus, exacerbating economic losses [5,7]. Naturally occurring low pathogenic (avirulent) strains of THEV have been isolated; these strains cause subclinical infections but retain immunosuppressive effects. Since its isolation from a pheasant spleen, the Virginia Avirulent Strain (VAS) has been effectively used as a live vaccine despite the immunosuppressive side effects. However, the vaccinated birds are rendered more susceptible to opportunistic infections and death than unvaccinated cohorts [4,5,8,9,10].

**Figure 1:**
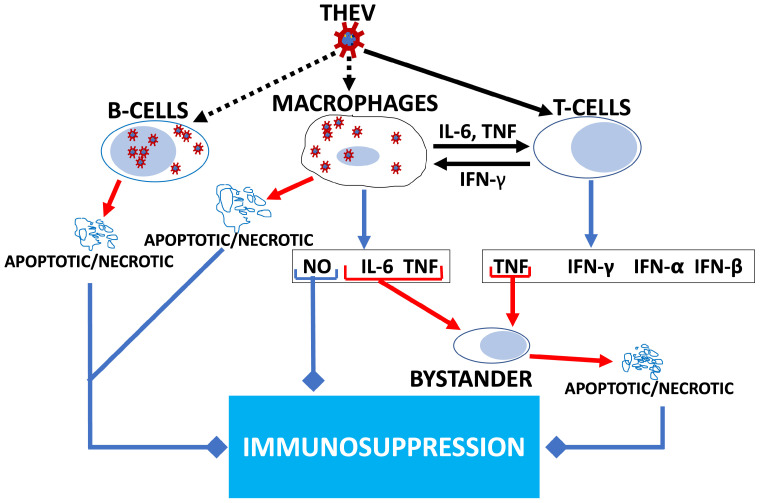
Model of THEV-induced immunosuppression in turkeys. THEV infection of target cells is indicated with black dotted arrows. Black unbroken arrows indicate cell activation. Red arrows indicated signals leading to cell death (apoptosis/necrosis). Blue arrows indicate all cytokines released by the cell. Blue arrows with square heads indicated an event leading to IMS. Adapted from Rautenschlein et al. [8].

It is well established that THEV primarily infects and replicates in turkey B-cells of the bursa and spleen and, to a lesser extent, in macrophages, inducing apoptosis and necrosis [6,8]. Consequently, a significant drop in the number of B-cells (specifically, IgM+ B-cells) and macrophages ensues along with increased T-cell counts with abnormal ratios of T-cell subpopulations (CD4+ and CD8+) [6,8,11]. Cell death seen in the infected B-cells and macrophages is generally proposed as the major cause of THEV-induced IMS as both humoral and cell-mediated immunity are impaired [5,8]. Immunopathogenesis via cytokines from T-cells and macrophages has also been suggested as a mechanism of apoptosis leading to IMS. It is thought that virus replication in the spleen attracts T-cells and peripheral blood macrophages, which results in T-cell activation by cytokines from activated macrophages and vice versa. The activated T-cells undergo clonal expansion and secrete type I (IFN-α and IFN-β) and type II (IFN-γ) interferons as well as tumor necrosis factor (TNF), while activated macrophages secrete interleukin 6 (IL-6), TNF, and nitric oxide (NO). These cytokines may further contribute to apoptosis and necrosis in bystander splenocytes, culminating in IMS [8,11] (Figure 1). However, the precise molecular mechanisms of THEV-induced IMS or the relevant intracellular signaling pathways are poorly understood [6]. Elucidating the specific mechanisms and pathways of THEV-induced IMS is a crucial step in THEV research as it could present a means of mitigating IMS.

Bulk mRNA sequencing (RNA-seq), a next-generation sequencing approach to transcriptomic studies, is a versatile, high throughput, and cost-effective technology that allows a broad survey of the entire transcriptome of a cell population, thereby uncovering the active genes and molecular pathways and processes. This technology has been leveraged in an ever-increasing number of studies to elucidate active cellular processes under a wide range of treatment conditions, including viral infections [12,13,14,15,16]. In RNA-seq studies, differentially expressed genes (DEGs) identified by contrasting pairs of different experimental conditions are key to unlocking the interesting biology or mechanism. Identified DEGs are typically used for functional enrichment analyses in large, curated knowledgebases such as gene ontology (GO) and Kyoto Encyclopedia of Genes and Genomes (KEGG) pathways, which connect genes to specific biological processes, functions, and pathways, shedding light on the biological question under study [17,18].

To our knowledge, no study has used RNA-seq to elucidate the molecular mechanisms and pathways leading to THEV-induced IMS. To effectively counteract the immunosuppressive effect of the vaccine, it is essential to unravel the host cell processes/pathways influenced by the virus to bring about IMS. In this study, we present the first transcriptomic profile of THEV-infected cells using paired-end bulk RNA-seq in a turkey B-cell line (MDTC-RP19), highlighting key host genes, cellular/molecular processes, and pathways affected during a THEV time course infection. We specifically focus on cellular processes related to cell survivability that can elucidate THEV-induced IMS.

## 2. Materials and Methods

### 2.1. Cell Culture and Infection

The turkey B-cell line (MDTC-RP19, ATCC CRL-8135) was grown as a suspension culture in 1:1 complete Leibovitz’s L-15/McCoy’s 5A medium with 10% fetal bovine serum (FBS), 20% chicken serum (ChS), 5% tryptose phosphate broth (TPB), and 1% antibiotic solution (100 U/mL penicillin and 100 μg/mL streptomycin), at 41 °C in a humidified atmosphere with 5% CO_2_. Infected cells were maintained in 1:1 serum-reduced Leibovitz’s L15/McCoy’s 5A media (SRLM) with 2.5% FBS, 5% ChS, 1.2% TPB, and 1% antibiotic solution. A commercially available THEV vaccine was purchased from Hygieia Biological Labs (VAS strain). The stock virus was titrated using an in-house qPCR assay with titer expressed as genome copy number (GCN)/mL, similar to Mahshoub et al. [19]. Cells were THEV-infected or mock-infected in triplicates or duplicates, respectively, at a multiplicity of infection (MOI) of 100 GCN/cell, incubated at 41 °C for 1 h, and washed three times with phosphate-buffered saline (PBS) to remove unattached virus particles. At each time point (4, 12, 24, and 72 hpi), triplicate (THEV-infected), and duplicate (mock-infected) samples were harvested for total RNA extraction.

### 2.2. RNA Extraction and Sequencing

Total RNA was extracted from infected cells using the Thermofisher (Waltham, MA, USA) RNAqueous™-4PCR Total RNA Isolation Kit (which includes a DNase I digestion step) per manufacturer’s instructions. Agarose gel electrophoresis was performed to check RNA integrity. The RNA quantity and purity were initially assessed using Nanodrop, and RNA was used only if the A260/A280 ratio was 2.0 ± 0.05 and the A260/A230 ratio was >2 and <2.2. Extracted total RNA samples were sent to LC Sciences, Houston, TX, for poly-A-tailed mRNA sequencing. RNA integrity was checked with Agilent Technologies 2100 Bioanalyzer High Sensitivity DNA Chip, and samples with an RNA integrity number (RIN) < 7 were excluded. Poly(A) RNA-seq library was prepared following Illumina’s TruSeq stranded mRNA sample preparation protocol. Paired-end sequencing, generating 149 bp reads, was performed on the Illumina NovaSeq 6000 sequencing system. The paired-end 149 bp sequences obtained during this study and all expression data have been submitted to the Gene Expression Omnibus database, under accession no GSE286211.

### 2.3. Quality Control and Mapping Process

Sequencing reads were processed following a well-established protocol described by Pertea et al. [20], using Snakemake—version 7.32.4 [21], a popular workflow management system to drive the pipeline. Briefly, raw sequencing reads were trimmed with Cutadapt—version 1.10 [22], and the quality of trimmed reads was evaluated using the FastQC software, version 0.12.1 (Bioinformatics Group at the Babraham Institute, Cambridge, UK; www.bioinformatics.babraham.ac.uk, accessed 2 January 2025), achieving an overall Mean Sequence Quality (PHRED Score) of 36. Trimmed reads were mapped to the reference turkey (*Meleagris gallopavo*) genome file GCF_000146605.3_Turkey_5.1_genomic.fna.gz (accessed 2 January 2025) from NCBI (genome build: melGal5) (https://ftp.ncbi.nlm.nih.gov/genomes/all/GCF/000/146/605/GCF_000146605.3_Turkey_5.1/) (accessed 2 January 2025) with Hisat2—version 2.2.1 [20] using the accompanying gene transfer format (GTF) annotation file (GCF_000146605.3_Turkey_5.1_genomic.gtf.gz) (accessed 2 January 2025) to build a genomic index. Samtools—version 1.21 was used to convert the output Sequence Alignment Map (SAM) file to the Binary Alignment Map (BAM) format. The StringTie (v2.2.1) software [20], set to expression estimation mode was used to generate normalized gene expression estimates from the BAM files for genes in the reference GTF file after which the prepDE.py3 script was used to extract read count information from the StringTie gene expression files, providing an expression count matrix for downstream DEG analysis.

### 2.4. DEG Analysis and Functional Enrichment Analysis

DEG analysis between mock- and THEV-infected samples was performed using DESeq2 version 3.20 [23], which employs a Negative Binomial distribution model for determining statistical significance when comparing read counts from multiple replicates. Genes with (FDR)-adjusted *p*-value ≤ 0.05 were considered as differentially expressed. The sequencing data (FASTQ files), expression count matrix, and DEG analysis results from DESeq2 are deposited at NCBI Gene Expression Omnibus under accession number GSE286211. The functional profiling of DEGs (GO and KEGG analyses) was performed based on GO databases and KEGG databases using DAVID and the R package (version 4.4.2) gprofiler2 [24] with *M. gallopavo* as the reference organism. Results with *P*_adjusted_-value ≤ 0.05 were included as functionally enriched. All visualization plots were made using ggplot2, pheatmap, and ggvenn R packages [25,26,27].

### 2.5. Validation of DEGs by Reverse Transcriptase Quantitative PCR (RT-qPCR)

The gene expression levels of representative DEGs (*APAF1*, *BMF*, *FADD*, *PDCD4*, *MADD*, *VCP*, *UFD1*, *EDEM1*, *EIF3D*, *EIF3M*, *RPL8*, *RPL10A*) were validated by quantification of relative mRNA levels with turkey *GAPDH* mRNA levels as the control gene. Briefly, the cells were infected, and RNA extracted as described for the RNA sequencing samples with three biological replicates at 12 and 24 hpi each for both THEV-infected or mock-infected samples. First-strand cDNA synthesis of total RNA was performed with an oligo-dT primer to amplify poly-A-tailed mRNA using SuperScript™ IV First-Strand Synthesis System. The parent RNA was digested using RNase H after cDNA synthesis was complete to ensure that only cDNA remained as the template for the RT-qPCR quantification. The RT-qPCR was performed with the PowerUp™ SYBR™ Green master mix from Applied Biosystems (Thermo Scientific, Waltham, MA, USA) with primers designed manually in the SnapGene software. The primers were checked for specificity using NCBI Nucleotide BLAST (https://blast.ncbi.nlm.nih.gov/Blast.cgi?PROGRAM=blastn) (accessed 1 January 2025) before use. All primers used in this study are listed in Supplementary Table S1. Relative mRNA levels were calculated by 2^−ΔΔCT^ method [28].

### 2.6. Statistical Analysis

Statistical analyses of the RT-qPCR results were performed using R (Version 4.3.3) with Student’s *t*-test for the comparison between two groups. A *p*-value ≤ 0.05 was considered statistically significant.

## 3. Results

### 3.1. Sequencing Results

To identify the host transcriptomic profile during THEV infection, we infected MDTC-RP19 cells with THEV or no virus (mock) in triplicates or duplicates, respectively, and harvested total RNA at 4, 12, 24, and 72 h post-infection (hpi). In the first 12 h, there was no discernible CPE. At 24 h, the CPE was very subtle but observable, and at 72 h, almost every cell was clearly swollen with numerous cytoplasmic vacuolation and granulation. Some cells were more than double the size of the mock-infected cells.

**Figure 2:**
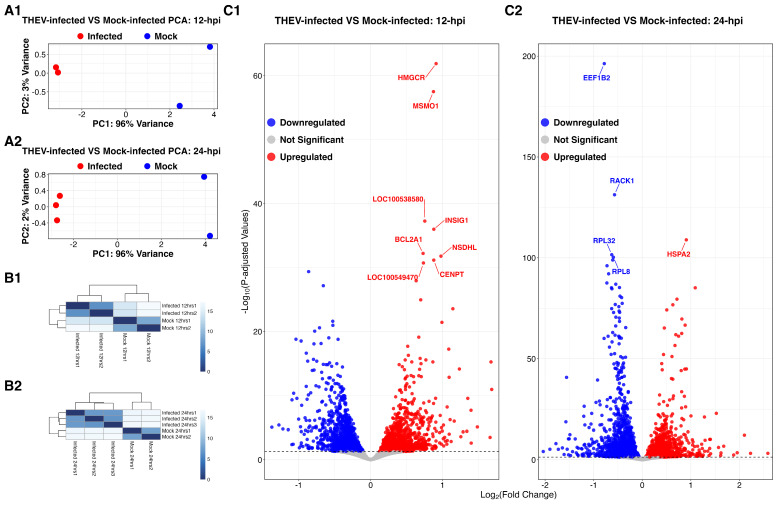
Principal component analysis (PCA) of turkey B-cells during THEV infection. At 12 hpi (**A1**), the results indicate that the first (PC1) and second (PC2) principal components account for 96% and 3% of the variation in the samples, respectively. Whereas PC1 and PC2 account for 96% and 2% of the variation, respectively at 24 hpi (**A2**). Poisson distance matrices illustrating the RNA-seq library integrity within treatment (infected versus mock) groups, with color scale representing the distances between biological replicates for both 12 hpi samples (**B1**) and 24 hpi samples (**B2**). Dark colors represent high correlation (similarity) between the samples involved. Volcano plots of DEGs between THEV-infected versus mock-infected cells at 12 hpi (**C1**) and 24 hpi (**C2**). Red, blue, and grey dots represent upregulated, downregulated, and non-significant genes, respectively, for both 12 hpi samples (**C1**) and 24 hpi samples (**C2**).

mRNAs extracted from mock- or THEV-infected cells were sequenced on the Illumina platform, yielding a total of 776.1 million raw reads (149 bp in length) across all samples (see Table 1 for sequencing statistics). After trimming low-quality reads, the remaining 742.8 million total paired-end trimmed reads (approximately 34.7–47.9 million reads per sample) were mapped to the reference genome of *M. gallopavo* obtained from the National Center for Biotechnology Information (NCBI). The percentage of reads that mapped to the host genome across all samples ranged from 32.4 to 89.2%. We observed that the fraction of reads that mapped to the host genome decreased while those mapping to the virus genome increased over the course of the infection as the viral infectious cycle progressed. Despite excellent quality scores at all time points (Table 1), DEGs identified at 4 and 72 hpi did not yield any statistically significant results in the downstream functional enrichment analyses (GO term and KEGG pathway analysis) and they were excluded from all subsequent analyses. In the remaining 12 and 24 hpi samples, a high consistency was observed between biological replicates (Figure 2). One biological replicate from the 12 h time point did not pass the RNA integrity quality control and was not sequenced.

### 3.2. DEGs of THEV-Infected Versus Mock-Infected Cells

**Figure 3:**
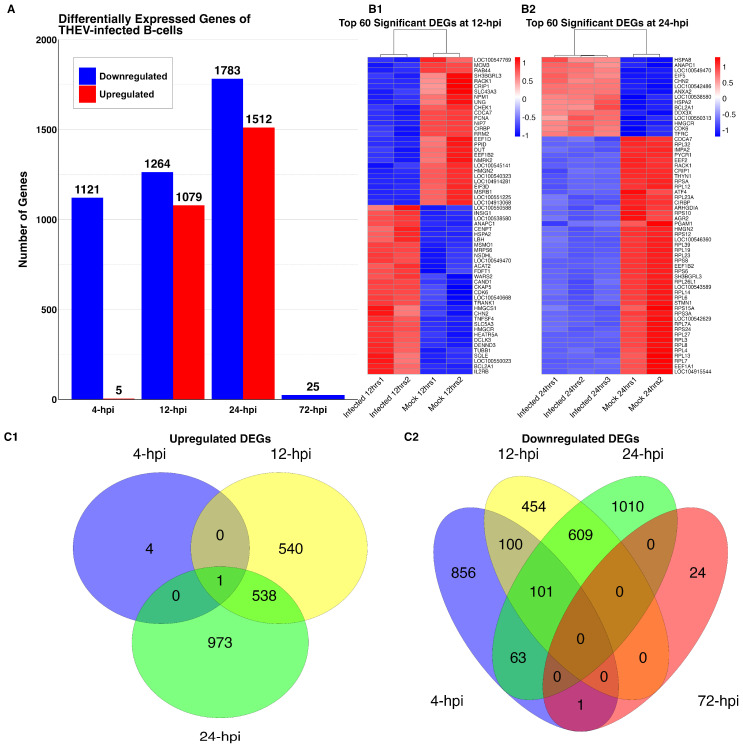
DEGs of THEV-infected versus mock-infected samples at different time points. (**A**) Bar plot of number DEGs identified. Red represents upregulated genes and blue represents downregulated genes. Heatmaps of scaled expression data (Z-scores) of DEGs. DEGs identified at 12 hpi are shown in (**B1**) and DEGs at 24 hpi in (**B2**). Venn diagrams showing the number of DEGs identified at different time points. For the upregulated genes (**C1**), the blue circle represents genes at 4 hpi, the yellow circle, 12 hpi, and the green circle, 24 hpi. For the downregulated genes (**C2**), the red circle represents genes at 72 hpi, while all the other time points retain the colors from (**C1**).

We quantified gene expression levels with the StringTie software [20] in Fragments per kilobase of transcript per million (FPKM) units. The differential expression analysis of DEGs was performed with the DESeq2 R package [23] which employs a negative binomial distribution model for determining statistical significance. Using a false discovery rate (FDR)-adjusted *p*-value cutoff ≤ 0.05 as the inclusion criteria, 2343 and 3295 genes from THEV-infected samples were identified as differentially expressed relative to their time-matched mock-infected samples at 12 hpi and 24 hpi, respectively. The results from the DEG analyses at 12 and 24 hpi have been deposited in NCBI Gene Expression Omnibus (http://www.ncbi.nlm.nih.gov/geo) (accessed 2 January 2025) under accession number GSE286211 with files named total_12hrsDEGs.csv.gz and total_24hrsDEGs.csv.gz, respectively. We compared THEV-infected samples relative to their time-matched mock-infected samples in identifying the significant DEGs and in the functional enrichment analyses. At 12 hpi (THEV-infected versus mock-infected), we found 1079 upregulated genes and 1264 downregulated genes, whereas 1512 genes were upregulated, and 1783 genes downregulated at 24 hpi (THEV-infected versus mock-infected) (Figure 2C1,C2 and Figure 3). The log_2_fold-change (FC) values at 12 hpi ranged between −1.4 and +1.7 for TMEM156 (Transmembrane Protein 156) and LIPG (Lipase G), respectively. At 24 hpi, the log_2_FC values ranged between −2.0 and +2.6 for C1QTNF12 (C1q And TNF Related 12) and KCNG1 (Potassium Voltage-Gated Channel Modifier Subfamily G Member 1), respectively.

### 3.3. Functional Enrichment Analyses (GO and KEGG Pathway Analyses)

Gene ontology (GO) enrichment analysis was performed for the DEGs determined at the 12 and 24 hpi timepoints with the DAVID (Database for Annotation, Visualization and Integrated Discovery; version 2021) online resource [29] and the gprofiler2 R package—version 0.2.3 [24], which outputs results according to the three branches of the GO directed acyclic graph—cellular components (CP), biological processes (BP), and molecular functions (MF). We compared THEV-infected samples relative to their time-matched mock-infected samples for each timepoint. Results with *P*_adjusted_-value ≤ 0.05 were considered functionally enriched. The GO enrichment analyses results at 12 hpi and 24 hpi showed significant overlaps among all three GO categories. At both time points, cellular breakdown processes were upregulated while cellular maintenance processes and structures were downregulated in all three GO categories (Table 2, Table 3, Table 4 and Table 5).

**Figure 4:**
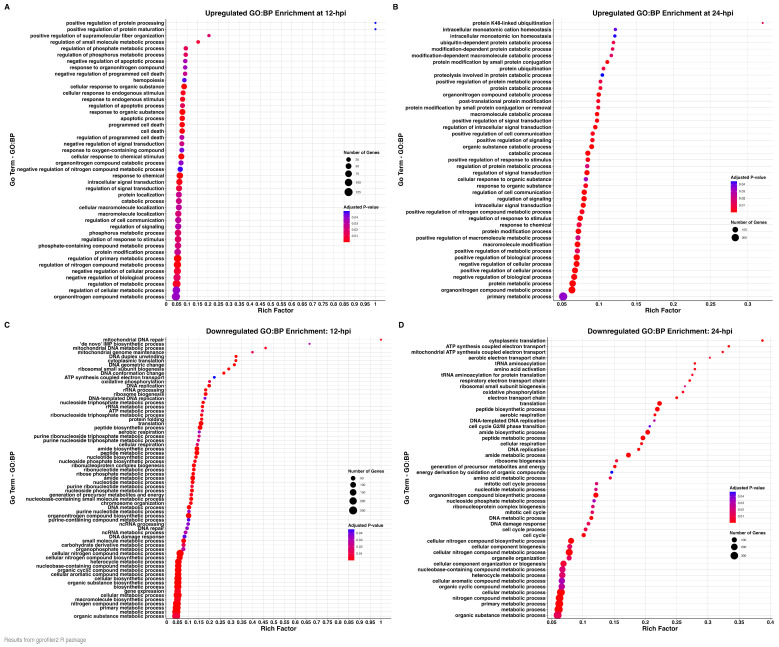
Dotplot of enriched gene ontology biological processes (BP). Significant BP GO terms identified for upregulated DEGs at 12 hpi and 24 hpi are shown in (**A**,**B**), respectively. Significant BP GO terms for downregulated DEGs at 12 hpi and 24 hpi are shown in (**C**,**D**), respectively. The *y*-axis indicates GO terms and the *x*-axis represents the rich factor, which indicates the ratio of the number of DEGs annotated to the term to the total number of genes annotated to the term. The diameter indicates the number of genes overlapping the gene ontology term, and the color indicates the enrichment *p*-value.

For upregulated DEGs at 12 hpi, we observed that the GO terms annotated under the BP category broadly cluster into apoptosis and autophagy, cellular metabolism (catabolic processes), sterol biosynthesis, response to stimuli, and protein processing (Figure 4A and Table 2). In the CC category, the GO terms relate primarily with cytoplasmic vacuolation, while in the MF category, they broadly fit under protein binding and kinase activity (Table 2). For downregulated DEGs at 12 hpi, the GO terms in the BP category generally fell under translation, protein biosynthesis and folding, ribosome biogenesis, nitrogen compound metabolism, nucleic acid synthesis, repair, metabolism, processing, replication, and energy metabolism. Also, immunoglobulin production and isotype switching were downregulated (Figure 4C and Table 3). In the CC category, GO terms broadly grouped into ribosome, mitochondria, respirosome, nucleus, and spliceosome, while in the MF category, they generally belong to translation regulator activity, protein folding chaperone, catalytic activity (acting on nucleic acids), and ATP hydrolysis activity (Table 3).

At 24 hpi, we found that the GO terms in the BP category for upregulated DEGs were associated with apoptosis and autophagy, lipid and sterol biosynthesis, catabolic process, protein ubiquitination and proteolysis, cell signaling, and cell metabolism. Additionally, host defense response and genes that negatively regulate cytokine production were upregulated (Figure 4B and Table 4). In the CC category, the GO terms were related to cytoplasmic vacuolation and the lysosome, similar to those identified at 12 hpi. In the MF category, the GO terms are grouped into protein ubiquitination activity, kinase and acyltransferase activity, and macromolecule binding activity (Table 4). GO terms for the downregulated DEGs were markedly similar to those at 12 hpi in all three GO categories. In the BP category, the GO terms broadly group into translation, peptide biosynthesis and folding, ribosome biogenesis, aerobic respiration and ATP synthesis, cell cycle process, and nucleic acid replication and processing (Figure 4D and Table 5). The GO terms in the CC category group under ribosome, mitochondrion, nucleus and chromosomes, while the MF category, the GO terms grouped into structural components of ribosome and translation regulator activity, catalytic activity acting on a nucleic acid and nucleic acid binding, aminoacyl-tRNA ligase activity, and NAD binding (Table 5).

KEGG pathway analysis on the DEGs was also performed using both the gprofiler2 R package [24] and the DAVID online resource. Both resources gave similar results, but the results from DAVID (Table 6) included more information than the gprofiler2 results (Table S2). The results from the KEGG pathway analysis were consistent with the GO results, revealing that generally, cell maintenance and upkeep pathways were downregulated while cell death and breakdown pathways were upregulated. We observed that cell maintenance pathways such as DNA replication and repair, ribosome biogenesis, spliceosome, and oxidative phosphorylation were downregulated at both 12 and 24 hpi. Pathways such as autophagy, response to virus (Influenza A), and steroid biosynthesis were upregulated at 12 hpi, which is similar to 24 hpi, where pathways such as autophagy, ubiquitin-mediated proteolysis, lysosome, protein processing in endoplasmic reticulum, and steroid biosynthesis were upregulated.

It is well-established that THEV induces cell death (apoptosis and necrosis) in infected B-cells, which is linked to THEV-induced IMS [8,11,30]. Hence, we were particularly interested in cellular processes and pathways associated with cell death and pathways that may affect the survival of the host B-cells, thereby accounting for THEV-induced IMS. We highlight the upregulated cell death (apoptosis and autophagy), ubiquitin-dependent endoplasmic reticulum [ER]-mediated protein degradation, and suppressed cell maintenance pathways as well as cytokine deregulation identified by our GO and KEGG analyses as the likely key aspects of THEV–host cell interaction relevant to THEV-induced IMS.

### 3.4. Cell Death and Breakdown Pathways Upregulated by THEV

Many virus families, including adenoviruses, herpesviruses, poxviruses, baculoviruses, parvoviruses, retroviruses, rhabdoviruses, paramyxoviruses, orthomyxoviruses, togaviruses, and picornaviruses, are known to trigger apoptosis in infected host cells either through direct viral protein action or the host antiviral response [31,32,33]. Our data show that apoptotic and autophagic pathways are upregulated during THEV infection, supporting previous findings of apoptosis and necrosis of THEV-infected cells [8,11,30]. For example, several proapoptotic members of the BCL2 (B-cell lymphoma 2) protein family, such as BCL2 antagonist/killer 1 (*BAK1*), BCL2 interacting protein 3 like (*BNIP3L*), BCL2 interacting protein 3 (*BNIP3*), and Bcl2 modifying factor (*BMF*), were upregulated. Additionally, Fas cell surface death receptor (*FAS*), Fas-associated via death domain (*FADD*), MAP kinase-activating death domain (*MADD*), programmed cell death 4 (*PDCD4*), RB1 inducible coiled-coil 1 (*RB1CC1*), activating transcription factor 4 (*ATF4*), receptor-interacting serine/threonine kinase 1 (*RIPK1*), tumor necrosis factor receptor superfamily member 1B (*TNFRSF1B*), pro-apoptotic WT1 regulator (*PAWR*), and apoptotic peptidase activating factor 1 (*APAF1*), which are potent proapoptotic factors were upregulated at both time points. Interestingly, both the intrinsic (*BAK1*, *BNIP3L*, *BNIP3*, *BMF*, *RB1CC1*, *ATF4*, *PDCD4*, and *APAF1*) and extrinsic (*FAS*, *FADD*, *TNFRSF1B*, *MADD*, and *RIPK1*) apoptotic pathways were represented. Conversely, several anti-apoptotic proteins such as BCL2 apoptosis regulator (*BCL2*), BCL2 interacting protein 2 (*BNIP2*; interacts directly with human adenovirus E1B-19K protein), BCL2 related protein A1 (*BCL2A1*), and apoptosis inhibitor 5 (*API5*) were also upregulated. Thus, apoptosis and its regulation pathways are clearly upregulated; this highlights the host-virus tug-of-war also typical in Mastadenovirus infections. Moreover, several genes associated with autophagy, such as TNF receptor-associated factor 6 (*TRAF6*), autophagy-related 9A (*ATG9A*), unc-51 like autophagy activating kinase 2 (*ULK2*), and autophagy-related 4B cysteine peptidase (*ATG4B*), were upregulated.

### 3.5. Downregulation of Cell Maintenance Pathways

Previous studies of human adenoviruses have shown that forcibly transitioning the host cell cycle to the S phase during the early phase of infection is a prerequisite for a productive adenovirus infection [34]. For human adenoviruses, interaction of the viral E1A early proteins with the host pRb (retinoblastoma) protein releases the host transcription factor E2F, which activates genes required for S phase cell cycle induction. Viral E1A also binds the host transcriptional co-activator p300/CBP [34,35]. Our GO and KEGG pathway results showed that at 12 hpi, several key genes involved with cell cycle transition were upregulated. Notably, E1A binding protein p300 (*EP300*), cyclin genes (*CCND3*, *CCNG1*, *CCNG2*, *CDK6*), anaphase-promoting complex subunit 1 (*ANAPC1*), and cell division cycle 27 (*CDC27*) were upregulated. However, unlike the observation in Mastadenoviruses, the cell cycle regulation at 12 and 24 hpi seem complicated as some key cell cycle-related genes and general cell maintenance processes were concurrently downregulated.

We found that several essential cell maintenance processes whose suppression can trigger apoptosis were downregulated. Severe DNA damage is a known mechanism of apoptosis induction, called DNA damage-dependent apoptosis [36]. Repression of host RNA and protein synthesis is also strongly associated with apoptosis [37]. Several processes related to DNA and RNA synthesis, maintenance, and repair, such as nucleotide biosynthesis and metabolism, double-strand break repair, DNA excision repair, RNA biosynthesis, RNA processing, DNA replication, mitotic cell cycle process, protein–RNA complex organization, and DNA damage response, were downregulated at both time points. Notable genes identified include DNA ligase 1 (*LIG1*), X-ray repair cross-complementing 1 (*XRCC1*), cyclin-dependent kinase 1 and 2 (*CDK1*, *CDK2*), checkpoint kinase 1 (*CHEK1*), 8-oxoguanine DNA glycosylase (*OGG1*), BLM RecQ-like-helicase (*BLM*), BRCA1 DNA repair associated (*BRCA1*), and several RAD family proteins (*RAD21*, *RAD51*, *RAD51B*, *RAD51C*, *RAD54B*).

Protein synthesis-related processes, including ribosome biogenesis, rRNA processing, ribosome assembly, protein folding, translational initiation, protein maturation, ribosome and ribonucleoprotein complex formation, translation pre-initiation complex formation, and cytoplasmic translation, were significantly downregulated at both 12 and 24 hpi. Notable genes identified include eukaryotic translation initiation factors (*EIF1*, *EIF1AX*, *EIF3E* and *EIF3F*, *EIF3H*, *EIF3I*, *EIF3L* and *EIF3M*), biogenesis of ribosomes BRX1 (*BRIX1*), MCTS1 re-initiation and release factor (*MCTS1*), and ribosomal protein subunits (*RPL8*, *RPL10a*, *RPL11*, *RP12*, *RP13*, *RP14*, *RP15*, *RP18a*, *RP19*).

### 3.6. Endoplasmic Reticulum (ER) Stress Response During THEV Infection

**Figure 5:**
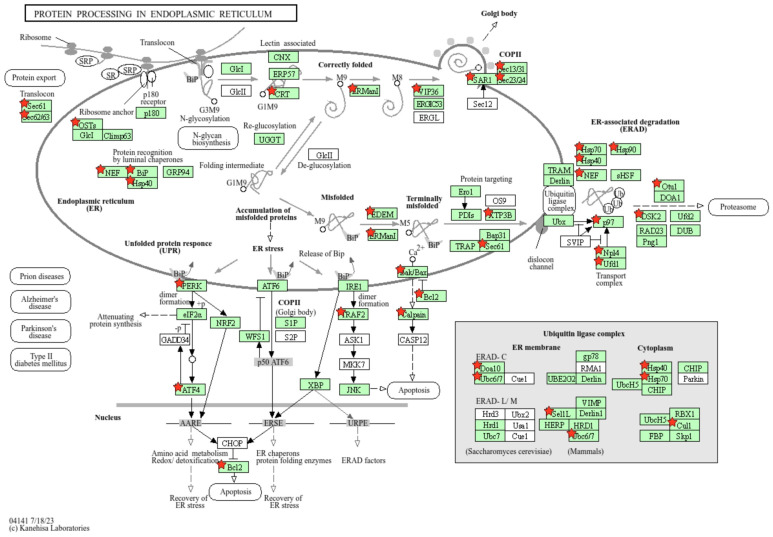
Upregulation of ER unfolded protein response (UPR). KEGG pathway analysis shows multiple key genes involved in the ER UPR were upregulated. All genes from our DEG list are annotated with the red star. Known turkey-specific pathways are colored green, while reference pathways are left uncolored. Notably, *ATF4*, *PERK*, *VCP* (*p97*), *TRAF2*, *UFD1*, and several BCL2 and heat shock proteins are upregulated. We see that the PERK branch of the UPR pathway linked to apoptosis is upregulated. Another pathway linked to apoptosis via *BAX* is shown, as well as the ERAD protein degradation pathway. Note that due to limited annotation of the host genome, a significant proportion of the DEGs were not recognized by the database; hence, not shown here. Figure generated from KEGG pathway analysis in DAVID [29].

Our KEGG pathway analysis (Table 4) showed that protein processing in the ER and ubiquitin-mediated proteolysis are significantly upregulated (Figure 5). The GO results (Table 4) showed that specifically, ER stress and the ER-associated protein degradation (ERAD) pathway, a branch of the unfolded protein response (UPR), were upregulated during THEV infection, especially at 24 hpi. The ER is the major site for protein synthesis, folding and quality control, and sorting [38]. Upon ER stress or continued accumulation of unfolded proteins in the ER lumen, the UPR pathways are activated to restore ER homeostasis. The ERAD pathway, a ubiquitin-proteasome-dependent pathway, is a protein quality control system activated for the degradation of misfolded and unassembled proteins [38]. In our results, the THEV-infected samples showed significant increase in ERAD pathway effector proteins, such as valosin containing protein (*VCP*), ubiquitin recognition factor in ER associated degradation 1 (*UFD1*), ER degradation enhancing alpha-mannosidase like proteins 1 and 3 (*EDEM1*, *EDEM3*), cullin 1 (*CUL1*), and ubiquitin 1 (*UBQLN1*). Other genes related to other UPR pathways, such as *HSPA5* and *ATF4*, were also upregulated. Our KEGG pathway (Table S2) and GO (Figure 4B) results indicated a significant upregulation of ubiquitin-mediated proteolysis with other ubiquitination pathway proteins such as ubiquitin-conjugating enzymes (*UBE2J2*, *UBE2E3*, *UBE2Z*), ubiquitin-protein ligases (*UBE3A*, *UBE3B*), NPL4 homolog ubiquitin recognition factor (*NPLOC4*), and ubiquitin-like modifier activating enzyme 6 (*UBA6*) showing significant upregulation. Additionally, the heat shock family of chaperone proteins such as the DnaJ heat shock protein family (*HSP40*) members (*DNAJB11*, *DNAJB12*, *DNAJB2*, *DNAJC10*), heat shock protein family A (*HSP70*) members (*HSPA4L*, *HSPA5*, *HSPA8*), and heat shock protein 90 alpha family class A member 1 (*HSP90AA1*) were upregulated at 24 hpi. Moreover, the KEGG pathway analysis (Table 4) shows a significant upregulation in lysosome formation, lumen acidification, and lysosomal degradation, likely an indication of ER-to-lysosome-associated degradation. Taken together, these results suggest that THEV infection triggers significant ER-associated protein degradation, which may contribute to cell death and IMS.

### 3.7. Differential Expression of Cytokine and Cytokine Receptor-Encoding Genes

Our KEGG pathway results showed that a pathway similar to immune response to influenza A infection was upregulated at 12 hpi. Our GO analysis also identified terms such as regulation of lymphocyte activation and regulation of cytokine production as upregulated at both 12 and 24 hpi. Genes involved include *IL18*, *IL2RB*, *IL4R*, *IL5RA*, TNF receptor-associated factors (*TRAF2*, *TRAF3*, *TRAF6*, *TRAF7*, *TRAFD1*), TNF receptor superfamily members (*TNFRSF1B*, *TNFRSF8*, *TNFSF4*), interferon-induced with helicase C domain 1 (*IFIH1*), interferon-induced double-stranded RNA-activated protein kinase (*PKR*), and *CD80*. In contrast, cytokine inhibitors such as suppressors of cytokine signaling (*SOCS3* and *SOCS5*) were also upregulated at both 12 and 24 hpi and immunoglobulin production and isotype switching GO terms were downregulated at 12 hpi. This inconsistency is likely an indicator of the struggle between the virus and its host. While several cytokines were regulated by THEV, as in the proposed model of THEV immunopathogenesis (Figure 1), the cytokines in the model (IFN-α, IFN-β, IFN-γ TNF, and IL-6) were not significantly differentially expressed in our data. However, some of the differentially expressed cytokines and cytokine receptors (*TNFRSF8*, *TRAF7*) identified in this study are positive regulators of apoptosis; therefore, they may play a role in THEV-induced IMS.

### 3.8. Validation of DEGs by Reverse Transcriptase Quantitative PCR (RT-qPCR)

**Figure 6:**
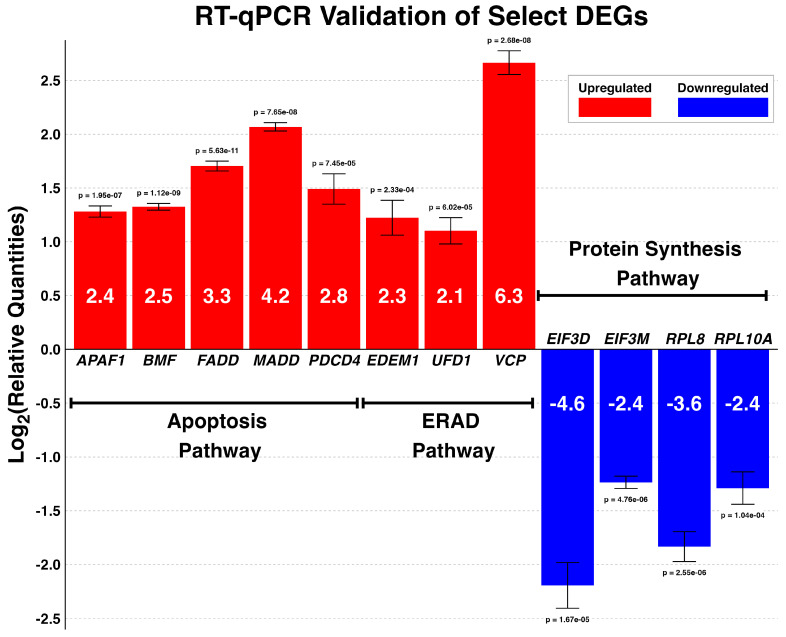
Validation of representative DEGs involved in apoptosis, protein synthesis, and ER-stress responses by RT-qPCR. MDTC-RP19 cells infected with THEV- or mock-infected were subjected to RT-qPCR analysis for the relative expression of the indicated DEGs at 24 hpi. *GAPDH* was used as the internal control. Data are expressed as the mean ± SD. All genes (THEV-infected) are statistically differentially expressed relative to their time-matched mock-infected counterparts based on Student’s t-test. The *p*-values are indicated on top of each bar, and the fold changes for each gene are indicated inside its corresponding bar.

To validate the RNA-seq results, 12 DEGs (eight upregulated and four downregulated) were selected for RT-qPCR. The DEGs were representative of apoptosis (*APAF1*, *BMF*, *FADD*, *MADD*, and *PDCD4*), ERAD and ubiquitination (*VCP*, *UFD1*, *EDEM1*), and ribosome biosynthetic (*EIF3D*, *EIF3M*, *RPL8*, *RPL10A*) pathways. As shown in Figure 6, the RT-qPCR results corroborate the RNA-seq results, further reinforcing the validity of the RNA-seq transcriptomic profile results. Although there was no inconsistency between the RNA-seq and RT-qPCR results in terms of gene regulations, the fold changes in the RT-qPCR results were consistently higher than observed in the RNA-seq results. Our RT-qPCR primers showed excellent target specificity; only one amplicon of the expected size was amplified, as shown by the melt curves and gel electrophoresis (Figure S1). According to our Student’s *t*-tests, the difference in gene expression levels in all the selected genes were statistically significant.

## 4. Discussion

THEV has a worldwide distribution, wreaking economic havoc on affected poultry farms, particularly due to its immunosuppressive trait, allowing secondary opportunistic infections to devastate turkey populations [4,6]. HE in turkeys causes more economic losses than any disease caused in other birds like chickens or pheasants [4]. While the current vaccine strain (VAS) has proven effective at preventing clinical HE in turkey poults, the retention of its immunosuppressive properties leaves some of the issues associated with economic losses unresolved. Elucidating the virus–host interactions leading to IMS is most pressing for not only the understanding of the viral infection and pathogenesis but also future antiviral therapy targets. Since both virulent and avirulent THEV cause IMS, but the avirulent are used as vaccine, we believe that studying VAS would be more expedient for understanding THEV vaccine-induced IMS.

Only one cell line (MDTC-RP19, also known as RP19) has been found to be capable of supporting THEV replication [39]. Thus, in this work, we establish the first transcriptomic profile of THEV infection in RP19 cells using paired-end RNA-seq. We attempted a multi-time point experimental design, but this being the first transcriptomic study of THEV infection, we faced some difficulties, including selecting our sampling time points based on the only study of THEV gene expression kinetics [40], leading to only 12 and 24 hpi providing useful data. In total, 2343 and 3295 DEGs were identified at 12 hpi and 24 hpi, respectively. At 12 hpi, 1079 genes were upregulated, and 1264 genes downregulated, whereas 1512 genes were upregulated, and 1783 genes were downregulated at 24 hpi. Being a non-model organism, a significant proportion of the host (*M. gallopavo*) genes are not annotated and not recognized by the databases used for functional enrichment analysis. Thus, the obtained results are likely sub-optimal in amount of detail relative to results from well-annotated and curated genomes of model organisms. The DEGs were related to multiple biological processes, all potentially playing a role in THEV infection, but the most relevant to our study are apoptosis (upregulated), ER stress-induced unfolded protein response (upregulated), cell maintenance processes (downregulated), and cytokine functions (deregulated). Furthermore, the RT-qPCR results validated the RNA-seq results. Collectively, this study may shed light on some significant aspects of THEV–host interactions, which may benefit further mechanistic delineation of the viral infection and induction of IMS and inform future development of anti-THEV strategies. The biological processes most relevant to THEV-induced IMS highlighted by this study are further discussed below.

Apoptosis is a key defense mechanism activated by cells in response to irreversible injury and virus infection to abrogate virus propagation. It is a formidable cellular defense network, non-specific to any virus family and, therefore an important problem for any infecting virus to overcome [31,32,33]. The human adenovirus E1A proteins are strong inducers of apoptosis. They bind host pRb and p300/CBP protein, inducing p53-mediated apoptosis, and can also sensitize infected cells to TNFα and TRAIL-induced apoptosis [34,35]. However, human adenoviruses have developed multiple distinct anti-apoptotic mechanisms to counter almost all cellular pro-apoptotic programs. For example, E1A blocks its own induction of p53-dependent apoptosis, and E1B proteins (E1B-19K and E1B-55K) counteract several types of apoptosis, including TNF-induced apoptosis [34,35]. Despite the rich arsenal of countermeasures, transcriptomic studies of human adenovirus infections suggest a complex set of virus–host interactions where both pro- and anti-apoptotic genes are turned on contemporaneously. For example, in human adenovirus 2 infection, both pro- and anti-apoptotic BCL2 family genes were stimulated [34]. Siadenoviruses, including THEV, are the smallest adenoviruses and therefore encode the fewest genes [10,41]. THEV encodes a mere 34 ORFs with no anti-apoptotic genes characterized [41]. In agreement with these findings, in our results, a strong signal indicative of apoptotic induction was observed. However, like Mastadenovirus infections, a complex relationship between pro and anti-apoptotic genes was observed. Pro-apoptotic genes such as *APAF1*, *BNIP3L*, *BMF*, *BAK1*, *RIPK1*, *FAS*, *FADD*, and *ATF* were upregulated in concert with the anti-apoptotic genes *BCL2*, *BNIP2*, *BCL2A1*, and *API5*. We speculate that this complex regulation is predictive of THEV possessing some anti-apoptotic genes but not sufficiently potent to thwart the cellular apoptotic response. It is also possible that using a naturally attenuated strain may account for the apparent balance in pro- and anti-apoptotic signals. Interestingly, pro-apoptotic genes in both intrinsic and extrinsic pathways were upregulated, possibly due to a concurrent stimulation of multiple apoptotic pathways or a positive feedback mechanism of one system activating the other. The specific mechanism of apoptosis induction remains elusive. Further studies designed to elucidate these fine details are warranted and would benefit future THEV therapeutics.

The ER serves many specialized functions, including biosynthesis and assembly of membrane and secretory proteins, calcium storage, and biosynthesis of lipids and sterols. It is also the site of protein folding and post-translational modifications and maintains stringent quality control systems, ensuring exported proteins are correctly folded and the degradation of unfolded or misfolded proteins [16,38,42]. Disruption of ER homeostasis or ER stress leads to the accumulation of incorrect proteins in the ER lumen, triggering the UPR. The UPR restores ER normality by transiently attenuating general protein synthesis, increasing the lumenal folding capacity, and the degradation of misfolded proteins through the ERAD pathway or autophagy [16,38,42,43]. However, if incorrect lumenal protein overload persists, the prolonged UPR will induce apoptosis known as ER stress-associated programmed cell death [42,43]. Many DNA and RNA viruses are reported to induce ER stress and UPR pathways during infection [16]. In our results, *ATF4* and PKR-like ER protein kinase (*PERK*), key proteins in the *PERK* branch of the UPR pathway, were upregulated. A myriad of ERAD pathway proteins (e.g., *VCP*, *UFD1*, *EDEM1*, *EDEM3*, *CUL1*, *UBQLN1*), ubiquitination system proteins (e.g., *UBE2J2*, *UBE2E3*, *UBE2Z*, *UBE3A*, *UBE3B*), and heat shock family of chaperone proteins (e.g., *HSPA5*, *HSP4L*, *HSPA8*, *HSP90AA1*) all showed increased expression according to our RNA-seq data with some validated with RT-qPCR. These data strongly suggest that THEV infection triggers the ER UPR pathways, leading to a massive decrease in protein synthesis and deregulation of sterol biosynthesis and ubiquitin-mediated proteolysis, all supported by our results. As noted above, a prolonged UPR activation leads to ER stress-associated programmed cell death via genes such as *ATF4* [42,43]. Thus, we suggest that the ER stress response likely plays a crucial role in THEV-induced IMS. Nonetheless, the mechanisms underlying the regulation of the UPR pathways by THEV remain to be clearly unraveled. Also, whether and how the ER stress response affects THEV infection and pathogenicity should be studied. Unsurprisingly, protein degradation was more evident at 24 hpi than at 12 hpi, reflecting the suggested two phases of UPR—Phase 1 allows the unfolded proteins time to refold without degradation, and Phase 2 degrades any proteins that have failed to fold [43].

In the proposed model of THEV immunopathogenesis by Rautenschlein et al. (Figure 1), while THEV directly induced cell death in infected cells, cytokines are responsible for extending cell death to bystander splenocytes [8]. However, the primary cytokines (IFN-α, IFN-β, IFN-γ TNF, IL-6, and NO) highlighted in the model were not significantly differentially expressed in our data. This may be explained by the fact that the model was proposed based on data from splenocytes of THEV-infected turkeys, which have the full complement of immune cells (T-cells, B-cells, macrophages) shown in the model and not solely from B-cell culture data as in this study. From the model, T-cells and macrophages are the principal producers of the effector cytokines; thus, there is agreement with our data that B-cells alone would poorly recapitulate the cytokine communication network. This may also explain the very few immune-associated biological processes in our data, as the B-cells may require cytokines from other immune cells such as macrophages and T-cells for optimal activation. Further transcriptomic studies with splenocytes would offer a wealth of insights regarding these ideas. It is also likely that cytokines may only play a dominant role in some aspects of THEV-infection, such as the clinical hemorrhage of the intestines but not the associated IMS since a study using the TNF-blocking drug (thalidomide) only prevented intestinal disease, not IMS [8]. While some of the upregulated cytokines and receptors in our results are positive apoptosis regulators (*TNFRSF8*, *TRAF7*), most of the cytokines are either anti-apoptotic (*TNFRSF1B*, *TRAF2*), boost host antiviral defense (*IL18*, *TNFSF4*, *PKR*, *TRAFD1*, *IFIH1*), or suppress cytokine signaling (*SOCS3*, *SOCS5*). Therefore, we speculate that a non-cytokine-mediated apoptotic process such as ER stress-associated programmed cell death is more likely to mediate the direct killing of infected cells. However, whether bystander cell death occurs and if it is cytokine-mediated, as suggested by Rautenschlein et al., are important questions that can be addressed with future transcriptomic studies in splenocytes.

By convention, the Mastadenovirus replication cycle is divided into two phases, an early and a late phase, separated by the onset of viral DNA replication [34,35]. Based on DNA microarray analysis, adenovirus type 2 (Ad2) infection has been divided into four stages. The first period is from 0 to 12 hpi, during which changes in cellular gene expression are likely to be triggered by the viral entry process. Most of the deregulated genes have functions linked to the inhibition of cell growth. Therefore, growth suppression is most likely the first response of the host cell to the incoming virus [34]. The second period covers the time from 12 to 24 hpi and follows activation of the immediate early E1A gene, which forcibly transitions the cell cycle to the S phase [34]. While the temporal changes in host gene expression for a THEV infection have not been characterized in prior studies, our data suggest that during the first 24 hpi, cell growth was suppressed. Cell maintenance processes involving nucleic acid and proteins were downregulated, according to our data. Protein synthesis-related processes, including ribosome biogenesis, rRNA processing, ribosome assembly, protein folding, translational initiation, protein maturation, and others, were heavily affected. Additionally, DNA and RNA synthesis, maintenance, and repair, such as nucleotide biosynthesis and metabolism, double-strand break repair, and DNA excision repair, were also repressed. As severe DNA damage leads to DNA damage-dependent apoptosis [36] and repression of RNA and protein synthesis is also strongly associated with apoptosis [37], these inhibitions may also play a role in THEV-induced IMS. Moreover, we speculate that the ER UPR may contribute partly to the severe repression of protein synthesis, as discussed above. An in-depth study of temporal changes in host gene expression during THEV infection would be invaluable in establishing if THEV follows the same pattern as Ad2.

## 5. Conclusions

THEV-induced IMS is a pressing concern for turkey farmers worldwide, causing substantial economic losses annually. In this study, we establish the cellular transcriptomic profile of THEV infection in turkey RP19 B-cells using paired-end RNA-seq, identifying 1079 upregulated genes and 1264 downregulated genes at 12 hpi, and 1512 upregulated genes and 1783 downregulated genes at 24 hpi. Our data suggest that several biological processes and pathways, including apoptosis, immune response, ER response to stress, ubiquitin-dependent proteolysis, and repression of essential cellular maintenance, are significant aspects of host cell response to THEV infection. All these processes are established apoptosis-inducing mechanisms; therefore, we believe that either one or synergistic interplay between multiple ones may mediate cell death of infected B-cells, leading to IMS. These findings provide the first insights into THEV–host interactions and may help advance the understanding of non-human adenoviral infection and pathogenesis, which may eventually inform the development of medical countermeasures for disease prevention and treatment.

## Figures and Tables

**Table 1:** Summary of sequencing, quality control, and mapping processes.

Sample	Raw Reads ^M^	Trimmed Reads ^M^	Mapped Reads ^M^	Uniquely Mapped Reads ^M^	Non-Uniquely Mapped Reads ^M^	Q20%	Q30%	GC Content (%)
I_12hrsS1 ^Inf^	40.6	39.0	34.7 (88.92%)	33.1 (84.78%)	1.6 (4.14%)	99.95	97.23	47.5
I_12hrsS3 ^Inf^	38.8	37.3	33.1 (88.78%)	31.7 (84.95%)	1.4 (3.83%)	99.95	97.53	47.5
I_24hrsS1 ^Inf^	42.7	41.0	36.2 (88.13%)	34.5 (84.2%)	1.6 (3.93%)	99.95	96.95	46.5
I_24hrsS2 ^Inf^	42.0	40.4	35.6 (88.1%)	33.9 (83.83%)	1.7 (4.27%)	99.94	97.05	46.5
I_24hrsS3 ^Inf^	40.5	38.9	34.2 (88.01%)	32.7 (84.12%)	1.5 (3.89%)	99.95	97.08	47.0
I_4hrsS1 ^Inf^	39.1	37.4	33 (88.16%)	31.2 (83.43%)	1.8 (4.73%)	99.93	97.04	48.5
I_4hrsS2 ^Inf^	41.3	39.6	35.3 (89.24%)	33.6 (84.92%)	1.7 (4.33%)	99.95	97.15	47.0
I_4hrsS3 ^Inf^	41.5	39.8	35.5 (89.2%)	33.2 (83.29%)	2.4 (5.91%)	99.95	97.11	47.5
I_72hrsS1 ^Inf^	41.2	39.8	28.3 (71.09%)	26.9 (67.7%)	1.3 (3.38%)	99.96	97.23	44.5
I_72hrsS2 ^Inf^	39.3	38.0	27 (71.11%)	25.8 (67.86%)	1.2 (3.25%)	99.96	97.34	44.5
I_72hrsS3 ^Inf^	39.9	37.1	28.3 (76.36%)	26.1 (70.3%)	2.2 (6.05%)	99.87	96.14	52.5
U_12hrsN1 ^Mk^	42.1	40.4	35.9 (88.72%)	34.1 (84.39%)	1.7 (4.33%)	99.95	97.04	47.5
U_12hrsN2 ^Mk^	41.0	39.3	34.7 (88.4%)	33.2 (84.53%)	1.5 (3.86%)	99.94	97.08	47.5
U_24hrsN1 ^Mk^	38.4	37.0	32.7 (88.46%)	31.4 (84.74%)	1.4 (3.72%)	99.96	97.48	47.5
U_24hrsN2 ^Mk^	39.9	38.4	34 (88.58%)	32.6 (84.96%)	1.4 (3.61%)	99.95	96.95	47.0
U_4hrsN1 ^Mk^	39.4	37.9	33.7 (88.9%)	32 (84.41%)	1.7 (4.49%)	99.96	97.36	47.0
U_4hrsN2 ^Mk^	37.6	34.7	22 (63.43%)	18.5 (53.18%)	3.6 (10.25%)	99.80	94.96	61.0
U_72hrsN1 ^Mk^	50.3	47.9	15.5 (32.4%)	11.7 (24.5%)	3.8 (7.9%)	99.88	96.54	56.0
U_72hrsN2 ^Mk^	40.5	38.9	34.5 (88.82%)	32.7 (84.14%)	1.8 (4.68%)	99.95	97.04	46.5

^M^ All values for number of reads are in millions; ^Inf^ These are infected samples indicated by the letter “I” and “S” in sample names; ^Mk^ These are mock-infected samples indicated by the letters “U” and “N” in sample names.

**Table 2:** Gene ontology analysis of significantly upregulated DEGs identified at 12 hpi.

GO Category	GO:Term	Fold Enrichment	Number of DEGs	*p*-Value (Adjusted)
Biological Process				
GO:BP	DNA-templated transcription	2.17	26	1.53 × 10^−2^
GO:BP	Alcohol biosynthetic process	3.77	19	3.45 × 10^−4^
GO:BP	Androgen receptor signaling pathway	10.03	5	3.18 × 10^−2^
GO:BP	Apoptotic process	2.75	47	6.09 × 10^−7^
GO:BP	Apoptotic signaling pathway	3.32	20	8.19 × 10^−4^
GO:BP	Appendage development	4.20	9	3.40 × 10^−2^
GO:BP	Appendage morphogenesis	4.59	8	4.22 × 10^−2^
GO:BP	Autophagy	2.59	23	4.43 × 10^−3^
GO:BP	Biological process involved in interspecies interaction between organisms	1.80	40	1.74 × 10^−2^
GO:BP	Biological regulation	1.14	517	8.20 × 10^−4^
GO:BP	Catabolic process	1.51	108	1.03 × 10^−3^
GO:BP	Cell cycle	1.68	72	1.34 × 10^−3^
GO:BP	Cell cycle phase transition	3.29	11	4.63 × 10^−2^
GO:BP	Cell cycle process	1.70	59	4.85 × 10^−3^
GO:BP	Cell death	2.85	51	4.99 × 10^−8^
GO:BP	Cell division	2.20	26	1.31 × 10^−2^
GO:BP	Cellular catabolic process	1.64	44	4.22 × 10^−2^
GO:BP	Cellular component disassembly	2.46	21	1.31 × 10^−2^
GO:BP	Cellular lipid biosynthetic process	9.36	7	3.16 × 10^−3^
GO:BP	Cellular lipid metabolic process	1.67	67	3.03 × 10^−3^
GO:BP	Cellular localization	1.45	145	4.34 × 10^−4^
GO:BP	Cellular macromolecule localization	1.58	104	4.21 × 10^−4^
GO:BP	Cellular metabolic process	1.23	307	8.52 × 10^−4^
GO:BP	Cellular response to biotic stimulus	3.21	12	3.47 × 10^−2^
GO:BP	Cellular response to chemical stimulus	1.56	60	2.49 × 10^−2^
GO:BP	Cellular response to decreased oxygen levels	4.86	8	3.18 × 10^−2^
GO:BP	Cellular response to hypoxia	5.02	8	2.79 × 10^−2^
GO:BP	Cellular response to lipid	2.66	20	8.44 × 10^−3^
GO:BP	Cellular response to lipopolysaccharide	3.56	11	2.92 × 10^−2^
GO:BP	Cellular response to molecule of bacterial origin	3.34	11	4.22 × 10^−2^
GO:BP	Cellular response to oxygen levels	5.02	9	1.27 × 10^−2^
GO:BP	Cellular response to oxygen-containing compound	1.92	33	1.98 × 10^−2^
GO:BP	Cellular response to stress	1.77	81	1.11 × 10^−4^
GO:BP	Cholesterol biosynthetic process	6.92	10	7.48 × 10^−4^
GO:BP	Cholesterol metabolic process	3.76	12	1.20 × 10^−2^
GO:BP	Deadenylation-independent decapping of nuclear-transcribed mrna	14.33	5	8.47 × 10^−3^
GO:BP	Developmental growth	2.58	19	1.53 × 10^−2^
GO:BP	Embryo development	1.93	29	3.43 × 10^−2^
GO:BP	Embryonic morphogenesis	2.36	24	9.41 × 10^−3^
GO:BP	Endoderm development	6.42	8	7.66 × 10^−3^
GO:BP	Ergosterol biosynthetic process	12.77	7	5.94 × 10^−4^
GO:BP	Ergosterol metabolic process	12.77	7	5.94 × 10^−4^
GO:BP	Establishment of localization	1.24	195	2.46 × 10^−2^
GO:BP	Establishment of localization in cell	1.56	104	5.94 × 10^−4^
GO:BP	Establishment of protein localization	1.61	73	3.79 × 10^−3^
GO:BP	Establishment of protein localization to organelle	2.04	34	7.41 × 10^−3^
GO:BP	Establishment or maintenance of cell polarity	2.51	20	1.48 × 10^−2^
GO:BP	Extrinsic apoptotic signaling pathway	4.18	10	1.89 × 10^−2^
GO:BP	Gland development	3.06	16	9.41 × 10^−3^
GO:BP	Growth	2.58	19	1.53 × 10^−2^
GO:BP	Hemopoiesis	2.16	26	1.69 × 10^−2^
GO:BP	Homeostasis of number of cells	3.27	14	1.27 × 10^−2^
GO:BP	Intracellular lipid transport	5.02	8	2.79 × 10^−2^
GO:BP	Intracellular protein transport	2.00	50	5.94 × 10^−4^
GO:BP	Intracellular signal transduction	1.54	97	1.48 × 10^−3^
GO:BP	Intracellular transport	1.51	79	1.02 × 10^−2^
GO:BP	Intrinsic apoptotic signaling pathway	3.70	12	1.31 × 10^−2^
GO:BP	Limb development	4.20	9	3.40 × 10^−2^
GO:BP	Limb morphogenesis	4.59	8	4.22 × 10^−2^
GO:BP	Lipid biosynthetic process	1.94	46	1.94 × 10^−3^
GO:BP	Lipid metabolic process	1.53	79	7.41 × 10^−3^
GO:BP	Localization	1.24	219	1.25 × 10^−2^
GO:BP	mRNA transcription	7.80	7	7.79 × 10^−3^
GO:BP	Macroautophagy	2.98	18	5.47 × 10^−3^
GO:BP	Macromolecule catabolic process	1.76	60	1.77 × 10^−3^
GO:BP	Macromolecule localization	1.58	128	3.56 × 10^−5^
GO:BP	Macromolecule metabolic process	1.21	286	6.45 × 10^−3^
GO:BP	Macromolecule modification	1.43	138	9.09 × 10^−4^
GO:BP	Metabolic process	1.19	426	3.93 × 10^−4^
GO:BP	Mitotic cell cycle	1.94	47	1.70 × 10^−3^
GO:BP	Mitotic cell cycle phase transition	3.34	11	4.22 × 10^−2^
GO:BP	Mitotic cell cycle process	2.14	41	8.20 × 10^−4^
GO:BP	Motor neuron apoptotic process	10.03	5	3.18 × 10^−2^
GO:BP	Multicellular organismal-level homeostasis	2.48	22	9.41 × 10^−3^
GO:BP	Negative regulation of apoptotic process	2.37	36	4.54 × 10^−4^
GO:BP	Negative regulation of biological process	1.56	187	1.90 × 10^−7^
GO:BP	Negative regulation of biosynthetic process	1.73	68	1.03 × 10^−3^
GO:BP	Negative regulation of cellular biosynthetic process	1.74	68	8.52 × 10^−4^
GO:BP	Negative regulation of cellular metabolic process	1.79	80	9.46 × 10^−5^
GO:BP	Negative regulation of cellular process	1.59	174	1.90 × 10^−7^
GO:BP	Negative regulation of gene expression	2.16	40	8.52 × 10^−4^
GO:BP	Negative regulation of intracellular signal transduction	2.07	28	1.78 × 10^−2^
GO:BP	Negative regulation of macromolecule biosynthetic process	1.70	65	2.25 × 10^−3^
GO:BP	Negative regulation of macromolecule metabolic process	1.66	82	7.20 × 10^−4^
GO:BP	Negative regulation of metabolic process	1.70	91	1.10 × 10^−4^
GO:BP	Negative regulation of programmed cell death	2.35	37	4.29 × 10^−4^
GO:BP	Nitrogen compound transport	1.57	79	4.15 × 10^−3^
GO:BP	Nuclear transport	2.24	22	2.79 × 10^−2^
GO:BP	Nuclear-transcribed mRNA catabolic process, deadenylation-independent decay	14.33	5	8.47 × 10^−3^
GO:BP	Nucleobase-containing compound catabolic process	2.05	24	4.40 × 10^−2^
GO:BP	Nucleocytoplasmic transport	2.24	22	2.79 × 10^−2^
GO:BP	Nucleoside bisphosphate metabolic process	3.03	13	3.39 × 10^−2^
GO:BP	Organic hydroxy compound biosynthetic process	2.93	20	3.11 × 10^−3^
GO:BP	Organonitrogen compound metabolic process	1.29	260	3.93 × 10^−4^
GO:BP	Organophosphate metabolic process	1.59	65	1.09 × 10^−2^
GO:BP	Phosphate-containing compound metabolic process	1.63	145	1.11 × 10^−6^
GO:BP	Phosphorus metabolic process	1.63	146	1.11 × 10^−6^
GO:BP	Phosphorylation	1.80	74	1.96 × 10^−4^
GO:BP	Phytosteroid biosynthetic process	12.04	9	3.75 × 10^−5^
GO:BP	Phytosteroid metabolic process	12.04	9	3.75 × 10^−5^
GO:BP	Positive regulation of apoptotic process	2.85	24	9.34 × 10^−4^
GO:BP	Positive regulation of biological process	1.33	193	1.29 × 10^−3^
GO:BP	Positive regulation of catabolic process	2.30	27	6.45 × 10^−3^
GO:BP	Positive regulation of cell communication	1.55	55	4.22 × 10^−2^
GO:BP	Positive regulation of cellular biosynthetic process	1.44	72	4.95 × 10^−2^
GO:BP	Positive regulation of cellular metabolic process	1.56	98	8.52 × 10^−4^
GO:BP	Positive regulation of cellular process	1.34	173	1.83 × 10^−3^
GO:BP	Positive regulation of macromolecule metabolic process	1.46	100	5.97 × 10^−3^
GO:BP	Positive regulation of metabolic process	1.53	116	4.34 × 10^−4^
GO:BP	Positive regulation of programmed cell death	2.74	24	1.58 × 10^−3^
GO:BP	Positive regulation of signal transduction	1.62	51	2.85 × 10^−2^
GO:BP	Positive regulation of signaling	1.55	55	4.22 × 10^−2^
GO:BP	Primary metabolic process	1.22	380	1.08 × 10^−4^
GO:BP	Process utilizing autophagic mechanism	2.59	23	4.43 × 10^−3^
GO:BP	Programmed cell death	2.85	51	4.99 × 10^−8^
GO:BP	Protein catabolic process	1.66	42	4.40 × 10^−2^
GO:BP	Protein localization	1.58	104	4.21 × 10^−4^
GO:BP	Protein localization to organelle	1.90	52	1.15 × 10^−3^
GO:BP	Protein metabolic process	1.27	198	8.44 × 10^−3^
GO:BP	Protein modification process	1.55	138	3.56 × 10^−5^
GO:BP	Protein phosphorylation	2.33	61	9.07 × 10^−7^
GO:BP	Protein transport	1.58	62	1.45 × 10^−2^
GO:BP	Purine nucleoside bisphosphate metabolic process	3.03	13	3.39 × 10^−2^
GO:BP	Regulation of DNA-templated transcription	1.35	142	7.41 × 10^−3^
GO:BP	Regulation of RNA biosynthetic process	1.35	142	7.59 × 10^−3^
GO:BP	Regulation of RNA metabolic process	1.34	154	5.97 × 10^−3^
GO:BP	Regulation of anatomical structure morphogenesis	1.81	32	4.74 × 10^−2^
GO:BP	Regulation of apoptotic process	2.15	57	2.79 × 10^−5^
GO:BP	Regulation of autophagy	2.61	20	1.02 × 10^−2^
GO:BP	Regulation of biological process	1.15	497	5.83 × 10^−4^
GO:BP	Regulation of biosynthetic process	1.40	210	3.56 × 10^−5^
GO:BP	Regulation of catabolic process	2.01	47	8.20 × 10^−4^
GO:BP	Regulation of cell communication	1.36	120	1.31 × 10^−2^
GO:BP	Regulation of cell cycle	1.67	43	3.66 × 10^−2^
GO:BP	Regulation of cell cycle process	1.79	33	4.65 × 10^−2^
GO:BP	Regulation of cellular biosynthetic process	1.41	210	2.24 × 10^−5^
GO:BP	Regulation of cellular catabolic process	2.35	21	2.18 × 10^−2^
GO:BP	Regulation of cellular metabolic process	1.48	252	2.56 × 10^−8^
GO:BP	Regulation of cellular process	1.16	473	4.21 × 10^−4^
GO:BP	Regulation of cytokine production	2.21	23	2.61 × 10^−2^
GO:BP	Regulation of developmental process	1.59	73	4.88 × 10^−3^
GO:BP	Regulation of epithelial cell apoptotic process	5.02	8	2.79 × 10^−2^
GO:BP	Regulation of gene expression	1.38	201	1.06 × 10^−4^
GO:BP	Regulation of intracellular signal transduction	1.60	73	4.75 × 10^−3^
GO:BP	Regulation of leukocyte differentiation	3.00	13	3.64 × 10^−2^
GO:BP	Regulation of macromolecule biosynthetic process	1.39	205	6.25 × 10^−5^
GO:BP	Regulation of macromolecule metabolic process	1.42	248	9.05 × 10^−7^
GO:BP	Regulation of metabolic process	1.47	279	6.08 × 10^−9^
GO:BP	Regulation of mitotic cell cycle phase transition	2.34	18	4.74 × 10^−2^
GO:BP	Regulation of nucleobase-containing compound metabolic process	1.36	167	1.29 × 10^−3^
GO:BP	Regulation of phosphate metabolic process	1.79	37	2.89 × 10^−2^
GO:BP	Regulation of phosphorus metabolic process	1.79	37	2.89 × 10^−2^
GO:BP	Regulation of primary metabolic process	1.40	226	1.02 × 10^−5^
GO:BP	Regulation of programmed cell death	2.07	57	6.77 × 10^−5^
GO:BP	Regulation of protein metabolic process	1.50	59	4.97 × 10^−2^
GO:BP	Regulation of response to stimulus	1.39	137	3.49 × 10^−3^
GO:BP	Regulation of response to stress	1.91	46	2.54 × 10^−3^
GO:BP	Regulation of signal transduction	1.44	110	4.85 × 10^−3^
GO:BP	Regulation of signaling	1.37	121	1.25 × 10^−2^
GO:BP	Regulation of transcription by RNA polymerase II	1.41	111	7.98 × 10^−3^
GO:BP	Response to chemical	1.56	95	1.24 × 10^−3^
GO:BP	Response to lipid	2.41	25	5.91 × 10^−3^
GO:BP	Response to nitrogen compound	1.97	30	2.46 × 10^−2^
GO:BP	Response to organonitrogen compound	2.19	29	7.59 × 10^−3^
GO:BP	Response to oxygen-containing compound	1.90	44	4.16 × 10^−3^
GO:BP	Response to stress	1.44	112	3.79 × 10^−3^
GO:BP	Ribonucleoside bisphosphate metabolic process	3.03	13	3.39 × 10^−2^
GO:BP	Secondary alcohol biosynthetic process	7.67	13	1.58 × 10^−5^
GO:BP	Secondary alcohol metabolic process	3.70	14	4.85 × 10^−3^
GO:BP	Small molecule biosynthetic process	2.38	45	3.75 × 10^−5^
GO:BP	Small molecule metabolic process	1.44	93	1.24 × 10^−2^
GO:BP	Steroid biosynthetic process	3.96	16	8.52 × 10^−4^
GO:BP	Steroid metabolic process	2.54	18	2.46 × 10^−2^
GO:BP	Sterol biosynthetic process	7.24	13	2.79 × 10^−5^
GO:BP	Sterol metabolic process	3.86	15	1.83 × 10^−3^
GO:BP	Tissue development	1.58	51	4.22 × 10^−2^
GO:BP	Transport	1.24	183	3.18 × 10^−2^
GO:BP	Vesicle-mediated transport	1.51	80	9.41 × 10^−3^
Cellular Component				
GO:CC	Golgi apparatus	1.52	69	1.40 × 10^−2^
GO:CC	Bounding membrane of organelle	1.70	92	3.49 × 10^−5^
GO:CC	Chromatin	1.84	40	9.58 × 10^−3^
GO:CC	Chromosome	1.58	63	1.10 × 10^−2^
GO:CC	Cytoplasm	1.28	590	7.06 × 10^−17^
GO:CC	Cytoplasmic vesicle	1.57	88	1.12 × 10^−3^
GO:CC	Cytoplasmic vesicle membrane	1.69	41	2.85 × 10^−2^
GO:CC	Cytosol	1.69	166	6.96 × 10^−10^
GO:CC	Early endosome	2.11	22	3.70 × 10^−2^
GO:CC	Endomembrane system	1.48	200	3.53 × 10^−7^
GO:CC	Endoplasmic reticulum	1.54	86	2.07 × 10^−3^
GO:CC	Endosome	1.69	48	1.37 × 10^−2^
GO:CC	Endosome membrane	2.02	25	3.21 × 10^−2^
GO:CC	Intracellular anatomical structure	1.19	774	8.23 × 10^−20^
GO:CC	Intracellular membrane-bounded organelle	1.29	578	1.08 × 10^−16^
GO:CC	Intracellular organelle	1.23	655	7.52 × 10^−16^
GO:CC	Intracellular organelle lumen	1.51	135	4.91 × 10^−5^
GO:CC	Intracellular vesicle	1.55	88	1.62 × 10^−3^
GO:CC	Membrane-bounded organelle	1.26	595	7.52 × 10^−16^
GO:CC	Membrane-enclosed lumen	1.51	135	4.91 × 10^−5^
GO:CC	Nuclear lumen	1.55	119	6.06 × 10^−5^
GO:CC	Nucleoplasm	1.75	104	1.57 × 10^−6^
GO:CC	Nucleus	1.42	371	1.33 × 10^−13^
GO:CC	Organelle	1.21	666	2.49 × 10^−14^
GO:CC	Organelle lumen	1.51	135	4.91 × 10^−5^
GO:CC	Organelle membrane	1.59	154	3.53 × 10^−7^
GO:CC	Organelle subcompartment	1.56	65	1.21 × 10^−2^
GO:CC	Perinuclear region of cytoplasm	2.51	25	2.11 × 10^−3^
GO:CC	Phagophore assembly site	4.73	8	2.83 × 10^−2^
GO:CC	Protein–DNA complex	1.76	42	1.40 × 10^−2^
GO:CC	Spindle	2.04	25	2.85 × 10^−2^
GO:CC	Transcription regulator complex	1.99	30	1.40 × 10^−2^
GO:CC	Vacuole	1.82	34	2.73 × 10^−2^
GO:CC	Vesicle	1.51	95	1.94 × 10^−3^
GO:CC	Vesicle membrane	1.74	43	1.40 × 10^−2^
Molecular Function				
GO:MF	ATP binding	1.33	128	2.71 × 10^−2^
GO:MF	DNA-binding transcription factor binding	2.73	22	5.83 × 10^−3^
GO:MF	R-SMAD binding	10.31	5	4.03 × 10^−2^
GO:MF	RNA polymerase II-specific DNA-binding transcription factor binding	2.94	18	1.05 × 10^−2^
GO:MF	Adenyl nucleotide binding	1.30	133	4.38 × 10^−2^
GO:MF	Adenyl ribonucleotide binding	1.33	130	2.71 × 10^−2^
GO:MF	Binding	1.09	714	4.02 × 10^−4^
GO:MF	Enzyme binding	2.20	102	8.60 × 10^−11^
GO:MF	Enzyme regulator activity	1.50	82	1.38 × 10^−2^
GO:MF	Identical protein binding	2.07	54	1.96 × 10^−4^
GO:MF	Ion binding	1.19	325	1.05 × 10^−2^
GO:MF	Kinase activity	1.58	86	4.95 × 10^−3^
GO:MF	Kinase binding	2.14	35	5.83 × 10^−3^
GO:MF	Manganese ion binding	5.62	9	1.05 × 10^−2^
GO:MF	Molecular adaptor activity	1.90	73	5.57 × 10^−5^
GO:MF	Myosin phosphatase activity	4.64	9	2.71 × 10^−2^
GO:MF	Nuclear androgen receptor binding	12.88	5	1.83 × 10^−2^
GO:MF	Phosphotransferase activity, alcohol group as acceptor	1.59	80	5.83 × 10^−3^
GO:MF	Protein binding	1.27	427	8.52 × 10^−8^
GO:MF	Protein domain specific binding	2.51	25	5.83 × 10^−3^
GO:MF	Protein homodimerization activity	2.29	23	2.51 × 10^−2^
GO:MF	Protein kinase activity	1.59	67	1.20 × 10^−2^
GO:MF	Protein kinase binding	2.10	31	1.18 × 10^−2^
GO:MF	Protein serine/threonine kinase activity	1.68	44	4.27 × 10^−2^
GO:MF	Protein–macromolecule adaptor activity	1.98	67	5.57 × 10^−5^
GO:MF	Purine ribonucleoside triphosphate binding	1.28	148	4.38 × 10^−2^
GO:MF	Signaling adaptor activity	3.44	12	3.01 × 10^−2^
GO:MF	Small molecule binding	1.19	338	5.83 × 10^−3^
GO:MF	Transcription coregulator activity	1.93	38	1.18 × 10^−2^
GO:MF	Transcription factor binding	2.38	27	6.59 × 10^−3^
GO:MF	Transferase activity	1.30	176	1.05 × 10^−2^
GO:MF	Transferase activity, transferring phosphorus-containing groups	1.51	96	5.83 × 10^−3^

**Table 3:** Gene ontology analysis of significantly downregulated DEGs identified at 12 hpi.

GO Category	GO:Term	Fold Enrichment	Number of DEGs	*p*-Value (Adjusted)
Biological Process				
GO:BP	“de novo” AMP biosynthetic process	10.16	5	2.26 × 10^−2^
GO:BP	“de novo” IMP biosynthetic process	11.43	5	1.51 × 10^−2^
GO:BP	“de novo” XMP biosynthetic process	18.29	4	1.71 × 10^−2^
GO:BP	“de novo” post-translational protein folding	8.31	10	6.05 × 10^−5^
GO:BP	“de novo” protein folding	7.95	10	8.96 × 10^−5^
GO:BP	AMP biosynthetic process	7.84	6	1.71 × 10^−2^
GO:BP	ATP biosynthetic process	6.58	9	1.26 × 10^−3^
GO:BP	ATP metabolic process	4.29	19	1.86 × 10^−5^
GO:BP	ATP synthesis coupled electron transport	6.10	18	1.95 × 10^−7^
GO:BP	DNA damage response	2.35	69	7.26 × 10^−9^
GO:BP	DNA geometric change	4.33	9	2.22 × 10^−2^
GO:BP	DNA integrity checkpoint signaling	3.75	16	6.72 × 10^−4^
GO:BP	DNA metabolic process	2.74	82	3.18 × 10^−14^
GO:BP	DNA recombination	2.07	23	3.64 × 10^−2^
GO:BP	DNA repair	2.39	51	1.44 × 10^−6^
GO:BP	DNA replication	4.95	39	2.28 × 10^−14^
GO:BP	DNA replication checkpoint signaling	6.36	8	5.00 × 10^−3^
GO:BP	DNA replication initiation	5.23	8	1.71 × 10^−2^
GO:BP	DNA strand elongation	6.97	8	2.67 × 10^−3^
GO:BP	DNA strand elongation involved in DNA replication	7.32	8	1.93 × 10^−3^
GO:BP	DNA-templated DNA replication	5.29	33	8.34 × 10^−13^
GO:BP	DNA-templated DNA replication maintenance of fidelity	4.43	8	3.69 × 10^−2^
GO:BP	GMP biosynthetic process	9.15	6	8.32 × 10^−3^
GO:BP	GMP metabolic process	7.84	6	1.71 × 10^−2^
GO:BP	NADH dehydrogenase complex assembly	4.57	8	3.13 × 10^−2^
GO:BP	RNA biosynthetic process	2.55	135	4.64 × 10^−22^
GO:BP	RNA export from nucleus	3.77	13	4.16 × 10^−3^
GO:BP	RNA localization	3.69	22	2.39 × 10^−5^
GO:BP	RNA metabolic process	2.49	152	6.25 × 10^−24^
GO:BP	RNA modification	2.30	19	3.26 × 10^−2^
GO:BP	RNA processing	3.04	120	6.65 × 10^−26^
GO:BP	RNA splicing	2.51	33	1.35 × 10^−4^
GO:BP	RNA splicing, via transesterification reactions	2.70	28	2.22 × 10^−4^
GO:BP	RNA splicing, via transesterification reactions with bulged adenosine as nucleophile	2.70	28	2.22 × 10^−4^
GO:BP	RNA transport	3.70	18	2.32 × 10^−4^
GO:BP	XMP biosynthetic process	18.29	4	1.71 × 10^−2^
GO:BP	XMP metabolic process	18.29	4	1.71 × 10^−2^
GO:BP	Aerobic electron transport chain	5.97	16	2.15 × 10^−6^
GO:BP	Aerobic respiration	5.10	29	1.17 × 10^−10^
GO:BP	Amino acid activation	3.25	11	3.83 × 10^−2^
GO:BP	Biosynthetic process	2.25	377	3.04 × 10^−57^
GO:BP	Carbohydrate derivative biosynthetic process	1.64	49	2.14 × 10^−2^
GO:BP	Carbohydrate derivative metabolic process	1.49	69	2.28 × 10^−2^
GO:BP	Cell cycle	1.47	69	3.10 × 10^−2^
GO:BP	Cell cycle DNA replication	6.33	9	1.67 × 10^−3^
GO:BP	Cell cycle checkpoint signaling	3.59	20	1.16 × 10^−4^
GO:BP	Cell cycle process	1.50	57	4.88 × 10^−2^
GO:BP	Cellular biosynthetic process	2.29	329	4.05 × 10^−50^
GO:BP	Cellular component assembly	1.34	117	2.26 × 10^−2^
GO:BP	Cellular component biogenesis	1.83	182	5.57 × 10^−14^
GO:BP	Cellular component organization or biogenesis	1.26	310	9.53 × 10^−5^
GO:BP	Cellular metabolic process	1.77	482	3.42 × 10^−45^
GO:BP	Cellular process	1.10	827	3.50 × 10^−7^
GO:BP	Cellular respiration	4.50	29	2.81 × 10^−9^
GO:BP	Cellular response to stress	1.79	90	5.95 × 10^−6^
GO:BP	Chaperone cofactor-dependent protein refolding	7.84	9	3.37 × 10^−4^
GO:BP	Chaperone-mediated protein folding	5.03	11	1.45 × 10^−3^
GO:BP	Chromosome organization	2.15	38	6.51 × 10^−4^
GO:BP	Cytoplasmic translation	9.15	27	7.21 × 10^−17^
GO:BP	Cytoplasmic translational initiation	7.48	9	4.85 × 10^−4^
GO:BP	Double-strand break repair via break-induced replication	7.32	6	2.28 × 10^−2^
GO:BP	Electron transport chain	5.08	20	5.49 × 10^−7^
GO:BP	Energy derivation by oxidation of organic compounds	3.00	30	1.49 × 10^−5^
GO:BP	Establishment of RNA localization	3.70	18	2.32 × 10^−4^
GO:BP	Establishment of protein localization to mitochondrion	3.09	12	3.30 × 10^−2^
GO:BP	Establishment of protein localization to organelle	1.91	35	1.13 × 10^−2^
GO:BP	Formation of cytoplasmic translation initiation complex	11.64	7	3.80 × 10^−4^
GO:BP	Gene expression	3.07	289	4.61 × 10^−70^
GO:BP	Generation of precursor metabolites and energy	2.77	40	1.13 × 10^−6^
GO:BP	Immunoglobulin production involved in immunoglobulin-mediated immune response	5.82	7	2.28 × 10^−2^
GO:BP	Import into nucleus	2.69	15	3.01 × 10^−2^
GO:BP	Import into the mitochondrion	3.92	12	5.82 × 10^−3^
GO:BP	Isotype switching	9.15	5	3.13 × 10^−2^
GO:BP	mRNA metabolic process	2.21	47	4.07 × 10^−5^
GO:BP	mRNA processing	2.50	39	2.09 × 10^−5^
GO:BP	mRNA splicing, via spliceosome	2.70	28	2.22 × 10^−4^
GO:BP	Macromolecule biosynthetic process	2.69	313	8.24 × 10^−63^
GO:BP	Macromolecule metabolic process	1.79	466	6.20 × 10^−45^
GO:BP	Macromolecule methylation	2.81	14	3.04 × 10^−2^
GO:BP	Maturation of 5.8S rRNA	6.79	13	1.21 × 10^−5^
GO:BP	Maturation of LSU-rRNA	8.13	16	1.88 × 10^−8^
GO:BP	Maturation of LSU-rRNA from tricistronic rRNA transcript (SSU-rRNA, 5.8S rRNA, LSU-rRNA)	5.57	7	2.86 × 10^−2^
GO:BP	Maturation of SSU-rRNA	6.22	17	4.15 × 10^−7^
GO:BP	Maturation of SSU-rRNA from tricistronic rRNA transcript (SSU-rRNA, 5.8S rRNA, LSU-rRNA)	6.27	12	8.09 × 10^−5^
GO:BP	metabolic process	1.57	618	9.00 × 10^−47^
GO:BP	Mitochondrial ATP synthesis coupled electron transport	6.10	17	5.49 × 10^−7^
GO:BP	Mitochondrial DNA metabolic process	9.85	7	1.15 × 10^−3^
GO:BP	Mitochondrial DNA replication	9.15	5	3.13 × 10^−2^
GO:BP	Mitochondrial electron transport, NADH to ubiquinone	6.58	9	1.26 × 10^−3^
GO:BP	Mitochondrial gene expression	2.93	12	4.86 × 10^−2^
GO:BP	Mitochondrial genome maintenance	6.10	7	1.85 × 10^−2^
GO:BP	Mitochondrial respiratory chain complex I assembly	4.57	8	3.13 × 10^−2^
GO:BP	Mitochondrial transmembrane transport	3.70	18	2.32 × 10^−4^
GO:BP	Mitochondrial transport	3.47	22	6.14 × 10^−5^
GO:BP	Mitochondrion organization	2.51	39	1.97 × 10^−5^
GO:BP	Mitotic cell cycle	1.69	45	2.01 × 10^−2^
GO:BP	Mitotic cell cycle checkpoint signaling	3.13	13	2.10 × 10^−2^
GO:BP	Mitotic cell cycle process	1.71	36	4.39 × 10^−2^
GO:BP	Negative regulation of DNA metabolic process	3.43	12	1.71 × 10^−2^
GO:BP	Negative regulation of cell cycle	2.19	24	1.71 × 10^−2^
GO:BP	Negative regulation of cell cycle phase transition	2.49	20	1.22 × 10^−2^
GO:BP	Negative regulation of cell cycle process	2.26	21	2.46 × 10^−2^
GO:BP	Non-membrane-bounded organelle assembly	1.99	30	1.66 × 10^−2^
GO:BP	Nuclear DNA replication	6.33	9	1.67 × 10^−3^
GO:BP	Nuclear export	3.33	20	3.06 × 10^−4^
GO:BP	Nuclear transport	2.79	30	5.99 × 10^−5^
GO:BP	Nucleic acid biosynthetic process	2.57	142	1.38 × 10^−23^
GO:BP	Nucleic acid metabolic process	2.56	226	1.57 × 10^−39^
GO:BP	Nucleic acid transport	3.70	18	2.32 × 10^−4^
GO:BP	Nucleobase-containing compound biosynthetic process	2.57	178	2.25 × 10^−30^
GO:BP	Nucleobase-containing compound metabolic process	2.41	276	9.74 × 10^−45^
GO:BP	Nucleobase-containing compound transport	3.22	22	1.83 × 10^−4^
GO:BP	Nucleobase-containing small molecule metabolic process	2.04	53	7.60 × 10^−5^
GO:BP	Nucleocytoplasmic transport	2.79	30	5.99 × 10^−5^
GO:BP	Nucleoside monophosphate biosynthetic process	5.12	14	9.79 × 10^−5^
GO:BP	Nucleoside monophosphate metabolic process	4.57	14	3.08 × 10^−4^
GO:BP	Nucleoside phosphate biosynthetic process	2.50	31	2.80 × 10^−4^
GO:BP	Nucleoside phosphate metabolic process	2.00	46	4.85 × 10^−4^
GO:BP	Nucleoside triphosphate biosynthetic process	5.23	16	1.41 × 10^−5^
GO:BP	Nucleoside triphosphate metabolic process	3.95	27	2.73 × 10^−7^
GO:BP	Nucleotide biosynthetic process	2.53	31	2.28 × 10^−4^
GO:BP	Nucleotide metabolic process	2.10	46	1.68 × 10^−4^
GO:BP	Organelle organization	1.23	174	4.94 × 10^−2^
GO:BP	Organonitrogen compound biosynthetic process	2.74	170	3.69 × 10^−32^
GO:BP	Organonitrogen compound metabolic process	1.50	331	2.81 × 10^−14^
GO:BP	Oxidative phosphorylation	6.29	22	1.17 × 10^−9^
GO:BP	Positive regulation of gene expression	1.74	37	3.13 × 10^−2^
GO:BP	Positive regulation of signal transduction by p53 class mediator	13.06	5	8.24 × 10^−3^
GO:BP	Positive regulation of translation	3.59	11	2.11 × 10^−2^
GO:BP	Primary metabolic process	1.63	556	1.30 × 10^−44^
GO:BP	Protein folding	4.00	35	6.76 × 10^−10^
GO:BP	Protein import into nucleus	2.80	15	2.20 × 10^−2^
GO:BP	Protein localization to mitochondrion	3.01	12	4.05 × 10^−2^
GO:BP	Protein localization to nucleus	2.49	17	3.01 × 10^−2^
GO:BP	Protein maturation	2.16	42	2.32 × 10^−4^
GO:BP	Protein metabolic process	1.52	261	2.39 × 10^−11^
GO:BP	Protein stabilization	3.85	16	4.94 × 10^−4^
GO:BP	Protein targeting	2.11	22	3.80 × 10^−2^
GO:BP	Protein targeting to mitochondrion	3.66	12	1.06 × 10^−2^
GO:BP	Protein–RNA complex assembly	5.76	40	3.24 × 10^−17^
GO:BP	Protein–RNA complex organization	5.54	40	1.35 × 10^−16^
GO:BP	Protein-containing complex assembly	2.19	83	2.81 × 10^−9^
GO:BP	Protein-containing complex organization	1.95	112	1.17 × 10^−9^
GO:BP	Proton motive force-driven ATP synthesis	6.86	9	9.28 × 10^−4^
GO:BP	Purine nucleoside monophosphate biosynthetic process	4.57	8	3.13 × 10^−2^
GO:BP	Purine nucleoside triphosphate biosynthetic process	4.91	11	1.78 × 10^−3^
GO:BP	Purine nucleoside triphosphate metabolic process	3.69	21	4.37 × 10^−5^
GO:BP	Purine nucleotide metabolic process	1.76	33	4.40 × 10^−2^
GO:BP	Purine ribonucleoside monophosphate biosynthetic process	4.57	8	3.13 × 10^−2^
GO:BP	Purine ribonucleoside triphosphate biosynthetic process	5.03	11	1.45 × 10^−3^
GO:BP	Purine ribonucleoside triphosphate metabolic process	3.88	21	2.09 × 10^−5^
GO:BP	Purine ribonucleotide metabolic process	1.94	29	2.52 × 10^−2^
GO:BP	rRNA metabolic process	5.57	63	4.00 × 10^−27^
GO:BP	rRNA modification	5.14	9	7.66 × 10^−3^
GO:BP	rRNA processing	5.97	62	1.61 × 10^−28^
GO:BP	Regulation of DNA metabolic process	2.92	30	2.42 × 10^−5^
GO:BP	Regulation of DNA replication	6.23	16	1.18 × 10^−6^
GO:BP	Regulation of DNA strand elongation	8.31	5	4.39 × 10^−2^
GO:BP	Regulation of DNA-templated DNA replication	7.84	6	1.71 × 10^−2^
GO:BP	Regulation of G2/M transition of mitotic cell cycle	3.41	11	2.94 × 10^−2^
GO:BP	Regulation of apoptotic process	1.58	46	4.77 × 10^−2^
GO:BP	Regulation of apoptotic signaling pathway	2.29	20	2.86 × 10^−2^
GO:BP	Regulation of cell cycle	1.95	55	1.68 × 10^−4^
GO:BP	Regulation of cell cycle phase transition	2.42	30	6.83 × 10^−4^
GO:BP	Regulation of cell cycle process	2.18	44	1.16 × 10^−4^
GO:BP	Regulation of protein stability	3.41	19	3.76 × 10^−4^
GO:BP	Regulation of signal transduction by p53 class mediator	6.10	7	1.85 × 10^−2^
GO:BP	Regulation of translation	2.26	20	3.13 × 10^−2^
GO:BP	Respiratory electron transport chain	4.99	18	4.43 × 10^−6^
GO:BP	Response to stress	1.45	123	9.23 × 10^−4^
GO:BP	Ribonucleoprotein complex biogenesis	5.52	108	1.71 × 10^−47^
GO:BP	Ribonucleoside monophosphate biosynthetic process	4.88	12	8.23 × 10^−4^
GO:BP	Ribonucleoside monophosphate metabolic process	4.39	12	2.07 × 10^−3^
GO:BP	Ribonucleoside triphosphate biosynthetic process	5.45	14	5.12 × 10^−5^
GO:BP	Ribonucleoside triphosphate metabolic process	4.14	24	8.49 × 10^−7^
GO:BP	Ribonucleotide biosynthetic process	2.42	22	8.55 × 10^−3^
GO:BP	Ribonucleotide metabolic process	2.12	34	2.14 × 10^−3^
GO:BP	Ribose phosphate biosynthetic process	2.55	24	2.07 × 10^−3^
GO:BP	Ribose phosphate metabolic process	2.19	36	7.72 × 10^−4^
GO:BP	Ribosomal large subunit assembly	10.16	10	9.32 × 10^−6^
GO:BP	Ribosomal large subunit biogenesis	8.18	34	8.01 × 10^−20^
GO:BP	Ribosomal small subunit assembly	8.54	7	2.70 × 10^−3^
GO:BP	Ribosomal small subunit biogenesis	7.36	31	2.14 × 10^−16^
GO:BP	Ribosome assembly	8.13	20	6.51 × 10^−11^
GO:BP	Ribosome biogenesis	5.80	86	2.32 × 10^−39^
GO:BP	Small molecule metabolic process	1.50	106	8.46 × 10^−4^
GO:BP	Somatic diversification of immunoglobulins involved in immune response	9.15	5	3.13 × 10^−2^
GO:BP	Somatic recombination of immunoglobulin genes involved in immune response	9.15	5	3.13 × 10^−2^
GO:BP	tRNA aminoacylation	3.41	11	2.94 × 10^−2^
GO:BP	tRNA metabolic process	2.81	31	3.49 × 10^−5^
GO:BP	tRNA transport	14.63	4	3.24 × 10^−2^
GO:BP	Telomere maintenance	3.35	11	3.13 × 10^−2^
GO:BP	Telomere organization	3.19	11	4.28 × 10^−2^
GO:BP	Translation	6.31	110	7.08 × 10^−55^
GO:BP	Translational elongation	4.30	8	4.31 × 10^−2^
GO:BP	Translational initiation	5.78	12	1.79 × 10^−4^
GO:BP	Viral gene expression	18.29	5	1.45 × 10^−3^
GO:BP	Viral translation	18.29	4	1.71 × 10^−2^
Cellular Component				
GO:CC	90S preribosome	8.05	20	1.61 × 10^−11^
GO:CC	Arp2/3 protein complex	7.89	5	2.33 × 10^−2^
GO:CC	Ctf18 RFC-like complex	15.77	5	1.30 × 10^−3^
GO:CC	DNA replication preinitiation complex	9.46	5	1.18 × 10^−2^
GO:CC	INO80-type complex	5.68	6	2.68 × 10^−2^
GO:CC	Ino80 complex	7.89	5	2.33 × 10^−2^
GO:CC	MCM complex	7.28	5	3.08 × 10^−2^
GO:CC	Sm-like protein family complex	3.57	13	2.69 × 10^−3^
GO:CC	U2-type prespliceosome	6.31	6	1.77 × 10^−2^
GO:CC	U2-type spliceosomal complex	4.32	13	4.48 × 10^−4^
GO:CC	catalytic complex	1.63	136	2.38 × 10^−7^
GO:CC	Catalytic step 2 spliceosome	3.40	14	2.49 × 10^−3^
GO:CC	Chaperonin-containing T-complex	7.36	7	2.67 × 10^−3^
GO:CC	Chromatin	1.64	41	2.10 × 10^−2^
GO:CC	Chromosome	1.98	91	1.20 × 10^−8^
GO:CC	Cytochrome complex	5.10	7	1.77 × 10^−2^
GO:CC	Cytoplasm	1.29	685	9.10 × 10^−22^
GO:CC	Cytosol	2.09	237	4.82 × 10^−28^
GO:CC	Cytosolic large ribosomal subunit	10.51	40	1.85 × 10^−29^
GO:CC	Cytosolic ribosome	10.70	69	3.41 × 10^−52^
GO:CC	Cytosolic small ribosomal subunit	12.20	29	1.62 × 10^−23^
GO:CC	Endopeptidase complex	3.11	13	8.59 × 10^−3^
GO:CC	Eukaryotic 43S preinitiation complex	12.62	8	1.11 × 10^−5^
GO:CC	Eukaryotic 48S preinitiation complex	15.14	8	1.98 × 10^−6^
GO:CC	Eukaryotic translation initiation factor 3 complex	12.17	9	2.20 × 10^−6^
GO:CC	Eukaryotic translation initiation factor 3 complex, eIF3m	15.14	4	1.26 × 10^−2^
GO:CC	Exosome (RNase complex)	5.16	6	3.92 × 10^−2^
GO:CC	Fibrillar center	3.44	10	1.94 × 10^−2^
GO:CC	Inner mitochondrial membrane protein complex	4.50	29	6.05 × 10^−10^
GO:CC	Intracellular anatomical structure	1.26	951	6.67 × 10^−51^
GO:CC	Intracellular membrane-bounded organelle	1.39	719	2.65 × 10^−36^
GO:CC	Intracellular non-membrane-bounded organelle	1.85	361	2.45 × 10^−34^
GO:CC	Intracellular organelle	1.33	823	1.15 × 10^−41^
GO:CC	Intracellular organelle lumen	2.62	271	6.67 × 10^−51^
GO:CC	Large ribosomal subunit	8.79	52	1.03 × 10^−33^
GO:CC	Membrane-bounded organelle	1.34	729	1.48 × 10^−31^
GO:CC	Membrane-enclosed lumen	2.62	271	6.67 × 10^−51^
GO:CC	Mitochondrial envelope	2.71	68	3.51 × 10^−12^
GO:CC	Mitochondrial inner membrane	3.34	50	3.51 × 10^−12^
GO:CC	Mitochondrial intermembrane space	4.73	9	5.04 × 10^−3^
GO:CC	Mitochondrial large ribosomal subunit	4.73	13	1.79 × 10^−4^
GO:CC	Mitochondrial matrix	3.99	42	9.69 × 10^−13^
GO:CC	Mitochondrial membrane	2.58	60	8.01 × 10^−10^
GO:CC	Mitochondrial protein-containing complex	4.57	55	9.42 × 10^−20^
GO:CC	Mitochondrial proton-transporting ATP synthase complex	6.08	9	8.94 × 10^−4^
GO:CC	Mitochondrial proton-transporting ATP synthase complex, coupling factor F(o)	6.76	5	3.92 × 10^−2^
GO:CC	Mitochondrial respirasome	5.16	6	3.92 × 10^−2^
GO:CC	Mitochondrial ribosome	5.03	21	5.82 × 10^−8^
GO:CC	Mitochondrial small ribosomal subunit	6.06	8	2.67 × 10^−3^
GO:CC	Mitochondrion	2.46	177	2.97 × 10^−28^
GO:CC	Non-membrane-bounded organelle	1.85	362	1.14 × 10^−34^
GO:CC	Nuclear chromosome	3.03	25	3.48 × 10^−5^
GO:CC	Nuclear envelope	2.30	34	1.90 × 10^−4^
GO:CC	Nuclear lumen	2.49	221	4.84 × 10^−37^
GO:CC	Nuclear membrane	2.38	16	2.43 × 10^−2^
GO:CC	Nuclear pore	3.51	13	3.05 × 10^−3^
GO:CC	Nuclear protein-containing complex	2.21	137	2.38 × 10^−17^
GO:CC	Nucleolus	4.68	111	8.60 × 10^−42^
GO:CC	Nucleoplasm	1.94	133	3.47 × 10^−12^
GO:CC	Nucleus	1.53	462	4.75 × 10^−26^
GO:CC	Organellar large ribosomal subunit	4.73	13	1.79 × 10^−4^
GO:CC	Organellar ribosome	5.03	21	5.82 × 10^−8^
GO:CC	Organellar small ribosomal subunit	6.06	8	2.67 × 10^−3^
GO:CC	Organelle	1.30	829	4.21 × 10^−37^
GO:CC	Organelle envelope	2.56	101	8.81 × 10^−17^
GO:CC	Organelle envelope lumen	4.37	9	8.53 × 10^−3^
GO:CC	Organelle inner membrane	3.14	54	4.23 × 10^−12^
GO:CC	Organelle lumen	2.62	271	6.67 × 10^−51^
GO:CC	Organelle membrane	1.25	140	3.13 × 10^−2^
GO:CC	Oxidoreductase complex	4.27	14	2.31 × 10^−4^
GO:CC	Peptidase complex	2.70	15	1.18 × 10^−2^
GO:CC	Preribosome	8.06	43	6.98 × 10^−26^
GO:CC	Preribosome, large subunit precursor	8.60	10	1.27 × 10^−5^
GO:CC	Preribosome, small subunit precursor	7.10	6	1.05 × 10^−2^
GO:CC	Prespliceosome	6.31	6	1.77 × 10^−2^
GO:CC	Protein folding chaperone complex	7.33	12	4.35 × 10^−6^
GO:CC	Protein–DNA complex	1.89	52	2.33 × 10^−4^
GO:CC	Protein-containing complex	1.73	467	5.00 × 10^−40^
GO:CC	Proton-transporting ATP synthase complex	5.87	9	1.16 × 10^−3^
GO:CC	Proton-transporting ATP synthase complex, coupling factor F(o)	7.10	6	1.05 × 10^−2^
GO:CC	Proton-transporting two-sector ATPase complex	3.26	10	2.66 × 10^−2^
GO:CC	Replication fork	4.82	13	1.51 × 10^−4^
GO:CC	Respirasome	5.22	8	6.59 × 10^−3^
GO:CC	Respiratory chain complex	5.82	8	3.33 × 10^−3^
GO:CC	Ribonucleoprotein complex	5.28	181	7.13 × 10^−79^
GO:CC	Ribosomal subunit	9.31	90	8.18 × 10^−62^
GO:CC	Ribosome	8.45	100	9.07 × 10^−64^
GO:CC	Rough endoplasmic reticulum	3.88	8	3.13 × 10^−2^
GO:CC	Small nuclear ribonucleoprotein complex	3.65	11	7.47 × 10^−3^
GO:CC	Small ribosomal subunit	10.00	37	3.59 × 10^−26^
GO:CC	Small-subunit processome	8.20	26	1.78 × 10^−15^
GO:CC	sno(s)RNA-containing ribonucleoprotein complex	7.57	8	6.08 × 10^−4^
GO:CC	Spliceosomal complex	2.63	29	8.28 × 10^−5^
GO:CC	Spliceosomal snRNP complex	3.86	11	4.98 × 10^−3^
GO:CC	Spliceosomal tri-snRNP complex	4.88	8	9.58 × 10^−3^
GO:CC	Translation preinitiation complex	13.10	9	1.03 × 10^−6^
Molecular Function				
GO:MF	ATP hydrolysis activity	1.81	38	1.92 × 10^−2^
GO:MF	ATP-dependent activity, acting on DNA	2.75	20	4.94 × 10^−3^
GO:MF	ATP-dependent protein folding chaperone	4.92	15	1.11 × 10^−4^
GO:MF	DNA helicase activity	4.66	13	1.02 × 10^−3^
GO:MF	NADH dehydrogenase (ubiquinone) activity	9.68	6	7.91 × 10^−3^
GO:MF	RNA binding	3.20	181	1.08 × 10^−43^
GO:MF	Catalytic activity, acting on DNA	2.37	32	1.02 × 10^−3^
GO:MF	Catalytic activity, acting on RNA	2.07	41	1.27 × 10^−3^
GO:MF	Catalytic activity, acting on a nucleic acid	2.13	71	3.41 × 10^−7^
GO:MF	Catalytic activity, acting on a tRNA	2.83	19	4.94 × 10^−3^
GO:MF	Electron transfer activity	5.16	8	2.07 × 10^−2^
GO:MF	Heat shock protein binding	3.45	13	1.17 × 10^−2^
GO:MF	Helicase activity	2.66	21	4.94 × 10^−3^
GO:MF	Heterocyclic compound binding	1.23	180	4.54 × 10^−2^
GO:MF	Hydrolase activity, acting on acid anhydrides	1.66	71	1.87 × 10^−3^
GO:MF	Hydrolase activity, acting on acid anhydrides, in phosphorus-containing anhydrides	1.64	70	2.65 × 10^−3^
GO:MF	Identical protein binding	2.09	58	2.05 × 10^−5^
GO:MF	Isomerase activity	2.25	21	3.26 × 10^−2^
GO:MF	mRNA binding	2.26	35	1.02 × 10^−3^
GO:MF	Nucleic acid binding	1.74	292	1.36 × 10^−21^
GO:MF	Nucleoside phosphate binding	1.24	173	3.69 × 10^−2^
GO:MF	Nucleotide binding	1.24	173	3.69 × 10^−2^
GO:MF	Organic cyclic compound binding	1.43	438	2.05 × 10^−17^
GO:MF	Oxidoreductase activity	1.55	68	1.17 × 10^−2^
GO:MF	Oxidoreductase activity, acting on NAD(P)H	4.63	11	4.18 × 10^−3^
GO:MF	Oxidoreductase activity, acting on NAD(P)H, quinone or similar compound as acceptor	7.74	8	2.16 × 10^−3^
GO:MF	Oxidoreduction-driven active transmembrane transporter activity	6.77	7	1.26 × 10^−2^
GO:MF	Poly(U) RNA binding	9.68	5	3.33 × 10^−2^
GO:MF	Protein folding chaperone	4.01	17	2.99 × 10^−4^
GO:MF	Protein-folding chaperone binding	3.45	13	1.17 × 10^−2^
GO:MF	Proton transmembrane transporter activity	2.79	17	1.23 × 10^−2^
GO:MF	Pyrophosphatase activity	1.65	70	2.23 × 10^−3^
GO:MF	rRNA binding	7.82	21	8.21 × 10^−11^
GO:MF	Ribonucleoprotein complex binding	3.39	17	1.91 × 10^−3^
GO:MF	Ribonucleoside triphosphate phosphatase activity	1.57	61	1.65 × 10^−2^
GO:MF	Ribosome binding	3.99	13	3.66 × 10^−3^
GO:MF	Single-stranded DNA binding	3.64	16	1.61 × 10^−3^
GO:MF	Single-stranded DNA helicase activity	7.04	8	3.73 × 10^−3^
GO:MF	snoRNA binding	10.84	14	7.89 × 10^−9^
GO:MF	structural constituent of nuclear pore	5.42	7	3.82 × 10^−2^
GO:MF	structural constituent of ribosome	9.21	88	5.28 × 10^−59^
GO:MF	Structural molecule activity	2.69	109	8.75 × 10^−19^
GO:MF	Translation elongation factor activity	7.53	7	7.78 × 10^−3^
GO:MF	Translation factor activity, RNA binding	5.36	23	1.83 × 10^−8^
GO:MF	Translation initiation factor activity	5.58	15	2.21 × 10^−5^
GO:MF	Translation regulator activity	4.84	29	8.60 × 10^−10^
GO:MF	Translation regulator activity, nucleic acid binding	4.94	24	3.61 × 10^−8^
GO:MF	Unfolded protein binding	4.65	25	4.96 × 10^−8^

**Table 4:** Gene ontology analysis of significantly upregulated DEGs identified at 24 hpi.

GO Category	GO:Term	Fold Enrichment	Number of DEGs	*p*-Value (Adjusted)
Biological Process				
GO:BP	ERAD pathway	6.28	14	1.77 × 10^−5^
GO:BP	Alcohol biosynthetic process	3.22	22	3.23 × 10^−4^
GO:BP	Autophagy	2.33	28	3.55 × 10^−3^
GO:BP	Biosynthetic process	1.21	252	1.72 × 10^−2^
GO:BP	Carbohydrate derivative metabolic process	1.47	84	1.58 × 10^−2^
GO:BP	Catabolic process	1.71	165	3.58 × 10^−9^
GO:BP	Cell death	1.73	42	2.50 × 10^−2^
GO:BP	Cellular biosynthetic process	1.24	220	1.58 × 10^−2^
GO:BP	Cellular catabolic process	1.76	64	1.02 × 10^−3^
GO:BP	Cellular homeostasis	1.64	45	3.99 × 10^−2^
GO:BP	Cellular lipid biosynthetic process	7.89	8	1.81 × 10^−3^
GO:BP	Cellular lipid metabolic process	1.60	87	1.20 × 10^−3^
GO:BP	Cellular localization	1.44	196	1.35 × 10^−5^
GO:BP	Cellular macromolecule localization	1.51	135	1.34 × 10^−4^
GO:BP	Cellular metabolic process	1.25	422	7.24 × 10^−6^
GO:BP	Cellular response to stress	1.69	105	1.87 × 10^−5^
GO:BP	Cellular response to topologically incorrect protein	2.87	13	4.82 × 10^−2^
GO:BP	Chaperone-mediated protein folding	3.70	10	3.73 × 10^−2^
GO:BP	Chemical homeostasis	1.61	54	2.28 × 10^−2^
GO:BP	Cholesterol biosynthetic process	7.14	14	3.72 × 10^−6^
GO:BP	Cholesterol metabolic process	3.70	16	1.34 × 10^−3^
GO:BP	Cytosolic transport	2.78	16	2.09 × 10^−2^
GO:BP	Embryonic epithelial tube formation	3.89	10	2.70 × 10^−2^
GO:BP	Embryonic morphogenesis	2.03	28	2.34 × 10^−2^
GO:BP	Endocytosis	1.83	41	1.20 × 10^−2^
GO:BP	Epithelial tube formation	3.79	10	3.22 × 10^−2^
GO:BP	Ergosterol biosynthetic process	10.76	8	1.76 × 10^−4^
GO:BP	Ergosterol metabolic process	10.76	8	1.76 × 10^−4^
GO:BP	Establishment of localization	1.40	298	9.39 × 10^−8^
GO:BP	Establishment of localization in cell	1.60	145	2.78 × 10^−6^
GO:BP	Establishment of protein localization	1.63	100	1.68 × 10^−4^
GO:BP	Establishment of protein localization to organelle	1.85	42	7.96 × 10^−3^
GO:BP	Glycoprotein metabolic process	1.88	40	8.79 × 10^−3^
GO:BP	Heparan sulfate proteoglycan biosynthetic process	7.75	11	5.17 × 10^−5^
GO:BP	Homeostatic process	1.60	73	3.78 × 10^−3^
GO:BP	Intracellular monoatomic cation homeostasis	1.82	36	2.50 × 10^−2^
GO:BP	Intracellular monoatomic ion homeostasis	1.82	36	2.63 × 10^−2^
GO:BP	Intracellular pH reduction	6.12	12	1.68 × 10^−4^
GO:BP	Intracellular protein transport	2.03	69	6.74 × 10^−6^
GO:BP	Intracellular signal transduction	1.39	119	8.90 × 10^−3^
GO:BP	Intracellular transport	1.66	118	8.14 × 10^−6^
GO:BP	Lipid biosynthetic process	1.77	57	2.31 × 10^−3^
GO:BP	Lipid metabolic process	1.50	105	1.77 × 10^−3^
GO:BP	Localization	1.34	322	9.94 × 10^−7^
GO:BP	Lysosomal lumen acidification	10.57	5	2.34 × 10^−2^
GO:BP	Macroautophagy	2.32	19	4.03 × 10^−2^
GO:BP	Macromolecule catabolic process	1.95	90	5.00 × 10^−7^
GO:BP	Macromolecule localization	1.48	162	4.06 × 10^−5^
GO:BP	Macromolecule metabolic process	1.35	435	1.22 × 10^−10^
GO:BP	Macromolecule modification	1.62	212	2.68 × 10^−10^
GO:BP	Metabolic process	1.25	609	1.89 × 10^−10^
GO:BP	Modification-dependent macromolecule catabolic process	1.84	49	3.12 × 10^−3^
GO:BP	Modification-dependent protein catabolic process	1.84	49	2.99 × 10^−3^
GO:BP	Monoatomic cation homeostasis	1.75	39	2.96 × 10^−2^
GO:BP	Monoatomic ion homeostasis	1.75	40	2.65 × 10^−2^
GO:BP	Morphogenesis of embryonic epithelium	3.70	11	2.25 × 10^−2^
GO:BP	Negative regulation of biological process	1.33	216	5.68 × 10^−4^
GO:BP	Negative regulation of biosynthetic process	1.56	83	3.32 × 10^−3^
GO:BP	Negative regulation of cellular biosynthetic process	1.57	83	2.79 × 10^−3^
GO:BP	Negative regulation of cellular metabolic process	1.64	99	1.56 × 10^−4^
GO:BP	Negative regulation of cellular process	1.37	203	1.70 × 10^−4^
GO:BP	Negative regulation of cytokine production	3.15	13	2.50 × 10^−2^
GO:BP	Negative regulation of gene expression	2.15	54	3.27 × 10^−5^
GO:BP	Negative regulation of intracellular signal transduction	2.24	41	2.58 × 10^−4^
GO:BP	Negative regulation of macromolecule biosynthetic process	1.53	79	8.47 × 10^−3^
GO:BP	Negative regulation of macromolecule metabolic process	1.45	97	9.56 × 10^−3^
GO:BP	Negative regulation of metabolic process	1.55	112	3.27 × 10^−4^
GO:BP	Neural tube formation	4.03	9	4.08 × 10^−2^
GO:BP	Nitrogen compound transport	1.73	118	1.35 × 10^−6^
GO:BP	Organic hydroxy compound biosynthetic process	2.48	23	6.55 × 10^−3^
GO:BP	Organonitrogen compound catabolic process	1.84	100	1.01 × 10^−6^
GO:BP	Organonitrogen compound metabolic process	1.39	381	1.64 × 10^−10^
GO:BP	Peptidyl-amino acid modification	2.40	42	4.49 × 10^−5^
GO:BP	Peptidyl-serine modification	2.87	13	4.82 × 10^−2^
GO:BP	Peptidyl-threonine modification	5.28	10	3.48 × 10^−3^
GO:BP	Phosphate-containing compound metabolic process	1.31	158	1.20 × 10^−2^
GO:BP	Phospholipid biosynthetic process	2.03	24	4.91 × 10^−2^
GO:BP	Phospholipid metabolic process	1.71	39	4.17 × 10^−2^
GO:BP	Phosphorus metabolic process	1.31	160	9.82 × 10^−3^
GO:BP	Phosphorylation	1.42	79	4.67 × 10^−2^
GO:BP	Phytosteroid biosynthetic process	9.86	10	1.77 × 10^−5^
GO:BP	Phytosteroid metabolic process	9.86	10	1.77 × 10^−5^
GO:BP	Positive regulation of apoptotic process	2.19	25	1.78 × 10^−2^
GO:BP	Positive regulation of biological process	1.26	249	2.53 × 10^−3^
GO:BP	Positive regulation of catabolic process	2.38	38	1.68 × 10^−4^
GO:BP	Positive regulation of cell communication	1.53	74	1.13 × 10^−2^
GO:BP	Positive regulation of cellular process	1.24	217	1.54 × 10^−2^
GO:BP	Positive regulation of intracellular signal transduction	1.61	49	3.55 × 10^−2^
GO:BP	Positive regulation of macromolecule metabolic process	1.32	123	3.22 × 10^−2^
GO:BP	Positive regulation of metabolic process	1.38	142	3.34 × 10^−3^
GO:BP	Positive regulation of programmed cell death	2.10	25	2.71 × 10^−2^
GO:BP	Positive regulation of protein catabolic process	2.64	18	1.71 × 10^−2^
GO:BP	Positive regulation of protein metabolic process	1.67	50	1.78 × 10^−2^
GO:BP	Positive regulation of response to stimulus	1.43	90	2.09 × 10^−2^
GO:BP	Positive regulation of signal transduction	1.64	70	3.22 × 10^−3^
GO:BP	Positive regulation of signaling	1.53	74	1.13 × 10^−2^
GO:BP	Post-translational protein modification	1.73	75	4.24 × 10^−4^
GO:BP	Primary metabolic process	1.32	558	9.52 × 10^−14^
GO:BP	Process utilizing autophagic mechanism	2.33	28	3.55 × 10^−3^
GO:BP	Programmed cell death	1.73	42	2.50 × 10^−2^
GO:BP	Proteasomal protein catabolic process	2.36	42	6.24 × 10^−5^
GO:BP	Proteasome-mediated ubiquitin-dependent protein catabolic process	1.90	28	4.97 × 10^−2^
GO:BP	Protein catabolic process	2.07	71	2.36 × 10^−6^
GO:BP	Protein export from nucleus	4.55	8	4.17 × 10^−2^
GO:BP	Protein folding	2.40	26	3.87 × 10^−3^
GO:BP	Protein localization	1.51	135	1.30 × 10^−4^
GO:BP	Protein localization to organelle	1.53	57	4.32 × 10^−2^
GO:BP	Protein localization to vacuole	2.76	14	4.39 × 10^−2^
GO:BP	Protein maturation	1.70	41	3.55 × 10^−2^
GO:BP	Protein metabolic process	1.48	313	1.04 × 10^−10^
GO:BP	Protein modification by small protein conjugation	1.59	56	2.50 × 10^−2^
GO:BP	Protein modification by small protein conjugation or removal	1.73	73	5.68 × 10^−4^
GO:BP	Protein modification by small protein removal	2.51	18	2.60 × 10^−2^
GO:BP	Protein modification process	1.68	202	1.04 × 10^−10^
GO:BP	Protein phosphorylation	1.80	64	5.84 × 10^−4^
GO:BP	Protein transport	1.62	86	8.20 × 10^−4^
GO:BP	Protein ubiquitination	1.60	51	3.47 × 10^−2^
GO:BP	Proteoglycan biosynthetic process	3.85	13	4.99 × 10^−3^
GO:BP	Proteoglycan metabolic process	3.70	15	2.31 × 10^−3^
GO:BP	Proteolysis	1.45	101	6.55 × 10^−3^
GO:BP	Proteolysis involved in protein catabolic process	1.97	63	5.57 × 10^−5^
GO:BP	Regulation of apoptotic process	1.62	58	1.54 × 10^−2^
GO:BP	Regulation of autophagy	2.31	24	1.20 × 10^−2^
GO:BP	Regulation of catabolic process	2.09	66	5.48 × 10^−6^
GO:BP	Regulation of cell communication	1.33	158	7.93 × 10^−3^
GO:BP	Regulation of cellular catabolic process	2.07	25	3.36 × 10^−2^
GO:BP	Regulation of cellular metabolic process	1.26	290	8.57 × 10^−4^
GO:BP	Regulation of cellular pH	3.19	19	1.47 × 10^−3^
GO:BP	Regulation of cytokine production	2.27	32	2.03 × 10^−3^
GO:BP	Regulation of cytoplasmic pattern recognition receptor signaling pathway	3.89	10	2.70 × 10^−2^
GO:BP	Regulation of defense response	1.88	34	2.33 × 10^−2^
GO:BP	Regulation of intracellular pH	3.13	18	2.85 × 10^−3^
GO:BP	Regulation of intracellular signal transduction	1.60	99	3.58 × 10^−4^
GO:BP	Regulation of lysosomal lumen pH	8.07	6	1.86 × 10^−2^
GO:BP	Regulation of macromolecule metabolic process	1.22	291	3.83 × 10^−3^
GO:BP	Regulation of metabolic process	1.27	329	6.35 × 10^−5^
GO:BP	Regulation of pH	2.99	19	3.08 × 10^−3^
GO:BP	Regulation of primary metabolic process	1.20	262	2.50 × 10^−2^
GO:BP	Regulation of programmed cell death	1.58	59	2.12 × 10^−2^
GO:BP	Regulation of proteasomal protein catabolic process	2.60	16	3.55 × 10^−2^
GO:BP	Regulation of protein catabolic process	2.47	27	2.19 × 10^−3^
GO:BP	Regulation of protein metabolic process	1.60	85	1.41 × 10^−3^
GO:BP	Regulation of proteolysis involved in protein catabolic process	2.78	19	6.74 × 10^−3^
GO:BP	Regulation of response to stimulus	1.34	179	2.19 × 10^−3^
GO:BP	Regulation of response to stress	1.78	58	1.81 × 10^−3^
GO:BP	Regulation of signal transduction	1.41	146	1.27 × 10^−3^
GO:BP	Regulation of signaling	1.32	158	1.06 × 10^−2^
GO:BP	Response to chemical	1.49	123	6.61 × 10^−4^
GO:BP	Response to endoplasmic reticulum stress	3.24	23	1.76 × 10^−4^
GO:BP	Response to nitrogen compound	1.93	40	5.15 × 10^−3^
GO:BP	Response to organonitrogen compound	2.06	37	3.12 × 10^−3^
GO:BP	Response to stress	1.42	149	8.20 × 10^−4^
GO:BP	Response to topologically incorrect protein	3.00	15	1.61 × 10^−2^
GO:BP	Secondary alcohol biosynthetic process	7.40	17	6.60 × 10^−8^
GO:BP	Secondary alcohol metabolic process	3.50	18	8.20 × 10^−4^
GO:BP	Small molecule biosynthetic process	1.72	44	2.28 × 10^−2^
GO:BP	Steroid biosynthetic process	3.65	20	1.68 × 10^−4^
GO:BP	Steroid metabolic process	2.50	24	4.19 × 10^−3^
GO:BP	Sterol biosynthetic process	6.99	17	1.44 × 10^−7^
GO:BP	Sterol metabolic process	3.60	19	3.27 × 10^−4^
GO:BP	Sulfur compound biosynthetic process	2.75	18	1.13 × 10^−2^
GO:BP	Sulfur compound metabolic process	1.95	30	2.66 × 10^−2^
GO:BP	Tissue morphogenesis	2.10	24	3.43 × 10^−2^
GO:BP	Transport	1.41	283	9.39 × 10^−8^
GO:BP	Ubiquitin-dependent protein catabolic process	1.89	49	1.89 × 10^−3^
GO:BP	Vacuolar acidification	6.16	10	1.18 × 10^−3^
GO:BP	Vacuolar transport	2.47	23	7.10 × 10^−3^
GO:BP	Vacuole organization	2.24	20	4.33 × 10^−2^
GO:BP	Vesicle organization	1.89	29	4.52 × 10^−2^
GO:BP	Vesicle-mediated transport	1.66	119	8.14 × 10^−6^
Cellular Component				
GO:CC	ATPase complex	2.46	19	1.18 × 10^−2^
GO:CC	ATPase dependent transmembrane transport complex	3.96	11	6.39 × 10^−3^
GO:CC	Golgi apparatus	1.68	105	4.83 × 10^−6^
GO:CC	Golgi apparatus subcompartment	1.83	28	4.19 × 10^−2^
GO:CC	Golgi cisterna	3.11	12	2.23 × 10^−2^
GO:CC	Golgi membrane	2.28	45	1.13 × 10^−5^
GO:CC	Bounding membrane of organelle	1.89	141	1.37 × 10^−11^
GO:CC	Catalytic complex	1.30	130	2.23 × 10^−2^
GO:CC	Cation-transporting ATPase complex	4.14	11	4.36 × 10^−3^
GO:CC	Clathrin-coated vesicle	2.72	16	1.23 × 10^−2^
GO:CC	Coated vesicle	2.38	26	1.90 × 10^−3^
GO:CC	Cytoplasm	1.30	827	6.20 × 10^−27^
GO:CC	Cytoplasmic vesicle	1.65	128	5.29 × 10^−7^
GO:CC	Cytoplasmic vesicle membrane	1.85	62	9.07 × 10^−5^
GO:CC	Cytosol	1.58	214	4.32 × 10^−10^
GO:CC	Early endosome	1.94	28	2.07 × 10^−2^
GO:CC	Endocytic vesicle	2.26	18	3.61 × 10^−2^
GO:CC	Endomembrane system	1.68	315	1.99 × 10^−20^
GO:CC	Endoplasmic reticulum	1.88	145	8.54 × 10^−12^
GO:CC	Endoplasmic reticulum membrane	1.98	84	1.17 × 10^−7^
GO:CC	Endoplasmic reticulum subcompartment	1.98	85	9.98 × 10^−8^
GO:CC	Endosome	1.81	71	3.79 × 10^−5^
GO:CC	Endosome membrane	2.16	37	4.17 × 10^−4^
GO:CC	Intracellular anatomical structure	1.16	1043	5.44 × 10^−20^
GO:CC	Intracellular membrane-bounded organelle	1.30	805	3.83 × 10^−25^
GO:CC	Intracellular organelle	1.19	881	2.20 × 10^−16^
GO:CC	Intracellular organelle lumen	1.53	190	5.31 × 10^−8^
GO:CC	Intracellular protein-containing complex	1.47	79	1.13 × 10^−2^
GO:CC	Intracellular vesicle	1.63	128	1.01 × 10^−6^
GO:CC	Lysosomal membrane	3.03	32	1.47 × 10^−6^
GO:CC	Lysosome	2.62	52	2.58 × 10^−8^
GO:CC	Lytic vacuole	2.59	52	3.79 × 10^−8^
GO:CC	Lytic vacuole membrane	3.03	32	1.47 × 10^−6^
GO:CC	Membrane	1.12	612	2.43 × 10^−3^
GO:CC	Membrane microdomain	2.66	16	1.48 × 10^−2^
GO:CC	Membrane raft	2.69	16	1.35 × 10^−2^
GO:CC	Membrane-bounded organelle	1.26	822	4.43 × 10^−22^
GO:CC	Membrane-enclosed lumen	1.53	190	5.31 × 10^−8^
GO:CC	Nuclear body	1.81	33	2.23 × 10^−2^
GO:CC	Nuclear lumen	1.55	164	4.35 × 10^−7^
GO:CC	Nuclear outer membrane-endoplasmic reticulum membrane network	1.94	84	2.90 × 10^−7^
GO:CC	Nucleolus	1.62	46	2.25 × 10^−2^
GO:CC	Nucleoplasm	1.67	137	9.98 × 10^−8^
GO:CC	Nucleus	1.24	447	1.08 × 10^−6^
GO:CC	Organelle	1.17	893	9.33 × 10^−14^
GO:CC	Organelle lumen	1.53	190	5.31 × 10^−8^
GO:CC	Organelle membrane	1.82	244	1.57 × 10^−19^
GO:CC	Organelle subcompartment	1.93	111	1.94 × 10^−9^
GO:CC	Perinuclear region of cytoplasm	2.47	34	6.02 × 10^−5^
GO:CC	Protein-containing complex	1.13	366	4.13 × 10^−2^
GO:CC	Proton-transporting V-type ATPase complex	4.52	8	2.23 × 10^−2^
GO:CC	Vacuolar membrane	2.73	39	9.21 × 10^−7^
GO:CC	Vacuolar proton-transporting V-type ATPase complex	6.33	8	2.95 × 10^−3^
GO:CC	Vacuole	2.48	64	2.52 × 10^−9^
GO:CC	Vesicle	1.56	136	3.62 × 10^−6^
GO:CC	Vesicle membrane	1.94	66	9.55 × 10^−6^
Molecular Function				
GO:MF	Acyltransferase activity	1.63	72	7.45 × 10^−3^
GO:MF	Binding	1.07	924	1.02 × 10^−2^
GO:MF	Catalytic activity	1.16	522	1.47 × 10^−3^
GO:MF	Catalytic activity, acting on a protein	1.28	230	4.88 × 10^−3^
GO:MF	Enzyme binding	1.95	120	2.10 × 10^−9^
GO:MF	Identical protein binding	2.17	75	2.02 × 10^−7^
GO:MF	Kinase binding	2.08	45	1.47 × 10^−3^
GO:MF	Lipid binding	1.57	62	4.54 × 10^−2^
GO:MF	Manganese ion binding	4.71	10	2.12 × 10^−2^
GO:MF	Misfolded protein binding	7.40	10	9.76 × 10^−4^
GO:MF	Protein binding	1.24	551	4.61 × 10^−8^
GO:MF	Protein domain specific binding	2.12	28	3.51 × 10^−2^
GO:MF	Protein kinase binding	2.04	40	4.88 × 10^−3^
GO:MF	Steroid binding	3.20	14	3.61 × 10^−2^
GO:MF	Transferase activity	1.30	233	2.67 × 10^−3^
GO:MF	Ubiquitin-like protein ligase binding	2.50	19	4.88 × 10^−2^

**Table 5:** Gene ontology analysis of significantly downregulated DEGs identified at 24 hpi.

GO Category	GO:Term	Fold Enrichment	Number of DEGs	*p*-Value (Adjusted)
Biological Process				
GO:BP	“de novo” IMP biosynthetic process	8.46	5	3.63 × 10^−2^
GO:BP	“de novo” XMP biosynthetic process	13.53	4	3.42 × 10^−2^
GO:BP	2′-deoxyribonucleotide biosynthetic process	7.89	7	3.48 × 10^−3^
GO:BP	2′-deoxyribonucleotide metabolic process	5.26	7	3.23 × 10^−2^
GO:BP	ADP catabolic process	3.54	11	2.13 × 10^−2^
GO:BP	ADP metabolic process	3.46	11	2.38 × 10^−2^
GO:BP	ATP biosynthetic process	5.95	11	2.49 × 10^−4^
GO:BP	ATP metabolic process	3.84	23	4.27 × 10^−6^
GO:BP	ATP synthesis coupled electron transport	6.26	25	1.40 × 10^−11^
GO:BP	DNA damage response	2.04	81	1.12 × 10^−7^
GO:BP	DNA integrity checkpoint signaling	2.95	17	5.00 × 10^−3^
GO:BP	DNA metabolic process	2.27	92	2.37 × 10^−11^
GO:BP	DNA recombination	2.13	32	3.19 × 10^−3^
GO:BP	DNA repair	2.28	66	5.27 × 10^−8^
GO:BP	DNA replication	3.29	35	9.38 × 10^−8^
GO:BP	DNA-templated DNA replication	3.44	29	8.52 × 10^−7^
GO:BP	DNA-templated DNA replication maintenance of fidelity	3.69	9	4.84 × 10^−2^
GO:BP	GMP biosynthetic process	6.76	6	2.73 × 10^−2^
GO:BP	L-amino acid biosynthetic process	3.09	13	2.14 × 10^−2^
GO:BP	L-amino acid metabolic process	2.46	28	9.12 × 10^−4^
GO:BP	RNA biosynthetic process	1.71	122	4.31 × 10^−7^
GO:BP	RNA metabolic process	1.89	156	1.03 × 10^−12^
GO:BP	RNA processing	1.85	99	2.56 × 10^−7^
GO:BP	RNA splicing	2.25	40	1.31 × 10^−4^
GO:BP	RNA splicing, via transesterification reactions	2.42	34	1.68 × 10^−4^
GO:BP	RNA splicing, via transesterification reactions with bulged adenosine as nucleophile	2.42	34	1.68 × 10^−4^
GO:BP	XMP biosynthetic process	13.53	4	3.42 × 10^−2^
GO:BP	XMP metabolic process	13.53	4	3.42 × 10^−2^
GO:BP	Aerobic electron transport chain	6.35	23	9.80 × 10^−11^
GO:BP	Aerobic respiration	5.33	41	7.30 × 10^−17^
GO:BP	Alpha-amino acid biosynthetic process	3.05	14	1.48 × 10^−2^
GO:BP	Alpha-amino acid metabolic process	2.16	29	5.33 × 10^−3^
GO:BP	Amino acid activation	4.80	22	1.38 × 10^−7^
GO:BP	Amino acid metabolic process	2.61	52	4.48 × 10^−8^
GO:BP	Biosynthetic process	1.78	404	6.23 × 10^−33^
GO:BP	Carbohydrate derivative metabolic process	1.42	89	1.91 × 10^−2^
GO:BP	Carboxylic acid metabolic process	1.76	78	6.39 × 10^−5^
GO:BP	Catabolic process	1.30	138	2.31 × 10^−2^
GO:BP	Cell cycle	2.28	145	5.63 × 10^−19^
GO:BP	Cell cycle DNA replication	4.68	9	1.22 × 10^−2^
GO:BP	Cell cycle checkpoint signaling	3.05	23	1.96 × 10^−4^
GO:BP	Cell cycle phase transition	2.83	14	2.68 × 10^−2^
GO:BP	Cell cycle process	2.35	121	1.51 × 10^−16^
GO:BP	Cell division	2.28	40	9.83 × 10^−5^
GO:BP	Cellular biosynthetic process	1.83	356	7.55 × 10^−31^
GO:BP	Cellular component assembly	1.51	179	8.36 × 10^−7^
GO:BP	Cellular component biogenesis	1.61	217	5.62 × 10^−11^
GO:BP	Cellular component disassembly	2.06	26	2.14 × 10^−2^
GO:BP	Cellular component organization	1.20	381	8.36 × 10^−4^
GO:BP	Cellular component organization or biogenesis	1.25	417	3.54 × 10^−6^
GO:BP	Cellular metabolic process	1.55	571	2.12 × 10^−32^
GO:BP	Cellular modified amino acid metabolic process	2.19	21	3.21 × 10^−2^
GO:BP	Cellular process	1.10	1121	1.42 × 10^−10^
GO:BP	Cellular respiration	4.93	43	2.46 × 10^−16^
GO:BP	Cellular response to stress	1.74	118	2.81 × 10^−7^
GO:BP	Centromere complex assembly	4.92	8	2.09 × 10^−2^
GO:BP	Chromatin organization	2.10	51	4.41 × 10^−5^
GO:BP	Chromatin remodeling	2.07	37	1.70 × 10^−3^
GO:BP	Chromosome organization	2.47	59	2.36 × 10^−8^
GO:BP	Chromosome segregation	2.49	47	1.02 × 10^−6^
GO:BP	Cytoplasmic translation	8.27	33	2.36 × 10^−20^
GO:BP	Cytoplasmic translational initiation	7.38	12	7.93 × 10^−6^
GO:BP	Deoxyribonucleotide biosynthetic process	6.37	8	3.90 × 10^−3^
GO:BP	Deoxyribonucleotide metabolic process	4.51	8	3.17 × 10^−2^
GO:BP	Deoxyribose phosphate biosynthetic process	7.89	7	3.48 × 10^−3^
GO:BP	Deoxyribose phosphate metabolic process	4.98	7	4.10 × 10^−2^
GO:BP	Double-strand break repair	2.15	31	3.48 × 10^−3^
GO:BP	Double-strand break repair via homologous recombination	2.29	20	2.53 × 10^−2^
GO:BP	Electron transport chain	5.64	30	1.03 × 10^−12^
GO:BP	Energy derivation by oxidation of organic compounds	3.40	46	5.23 × 10^−11^
GO:BP	Fatty acid beta-oxidation	3.57	14	3.37 × 10^−3^
GO:BP	Fatty acid oxidation	3.27	14	7.85 × 10^−3^
GO:BP	Formation of cytoplasmic translation initiation complex	9.84	8	1.51 × 10^−4^
GO:BP	Formation of translation preinitiation complex	9.66	5	2.30 × 10^−2^
GO:BP	Gene expression	2.26	288	1.04 × 10^−39^
GO:BP	Generation of precursor metabolites and energy	3.07	60	1.49 × 10^−12^
GO:BP	Glycolytic process	3.63	11	1.81 × 10^−2^
GO:BP	Import into the mitochondrion	3.14	13	1.89 × 10^−2^
GO:BP	Lipid oxidation	3.21	14	9.33 × 10^−3^
GO:BP	mRNA metabolic process	1.95	56	1.28 × 10^−4^
GO:BP	mRNA processing	2.14	45	1.28 × 10^−4^
GO:BP	mRNA splicing, via spliceosome	2.42	34	1.68 × 10^−4^
GO:BP	Macromolecule biosynthetic process	2.03	319	5.47 × 10^−35^
GO:BP	Macromolecule catabolic process	1.43	72	4.46 × 10^−2^
GO:BP	Macromolecule metabolic process	1.54	540	3.53 × 10^−29^
GO:BP	Maturation of LSU-rRNA	3.76	10	2.42 × 10^−2^
GO:BP	Meiosis I cell cycle process	2.43	16	4.50 × 10^−2^
GO:BP	Meiotic cell cycle	2.42	29	8.58 × 10^−4^
GO:BP	Meiotic cell cycle process	2.71	28	1.68 × 10^−4^
GO:BP	Meiotic nuclear division	2.71	24	8.57 × 10^−4^
GO:BP	Metabolic process	1.43	761	3.14 × 10^−36^
GO:BP	Microtubule cytoskeleton organization involved in mitosis	2.67	28	2.13 × 10^−4^
GO:BP	Microtubule-based process	1.44	85	1.63 × 10^−2^
GO:BP	Mitochondrial ATP synthesis coupled electron transport	6.37	24	2.94 × 10^−11^
GO:BP	Mitochondrial electron transport, NADH to ubiquinone	5.41	10	1.70 × 10^−3^
GO:BP	Mitochondrial electron transport, succinate to ubiquinone	13.53	4	3.42 × 10^−2^
GO:BP	Mitochondrial gene expression	3.07	17	3.37 × 10^−3^
GO:BP	Mitochondrial translation	3.64	14	2.78 × 10^−3^
GO:BP	Mitochondrial transmembrane transport	2.89	19	2.75 × 10^−3^
GO:BP	Mitochondrial transport	2.68	23	1.42 × 10^−3^
GO:BP	Mitochondrion organization	1.81	38	1.58 × 10^−2^
GO:BP	Mitotic DNA integrity checkpoint signaling	3.06	12	3.38 × 10^−2^
GO:BP	Mitotic cell cycle	2.64	95	5.25 × 10^−16^
GO:BP	Mitotic cell cycle checkpoint signaling	3.03	17	3.78 × 10^−3^
GO:BP	Mitotic cell cycle phase transition	2.87	14	2.38 × 10^−2^
GO:BP	Mitotic cell cycle process	2.68	76	9.49 × 10^−13^
GO:BP	Mitotic nuclear division	2.82	20	2.41 × 10^−3^
GO:BP	Mitotic sister chromatid segregation	2.92	19	2.39 × 10^−3^
GO:BP	Mitotic spindle organization	2.76	22	1.43 × 10^−3^
GO:BP	Negative regulation of cell cycle	2.03	30	1.10 × 10^−2^
GO:BP	Negative regulation of cell cycle phase transition	2.30	25	6.05 × 10^−3^
GO:BP	Negative regulation of cell cycle process	2.15	27	9.53 × 10^−3^
GO:BP	Non-membrane-bounded organelle assembly	2.40	49	1.65 × 10^−6^
GO:BP	Nuclear DNA replication	4.68	9	1.22 × 10^−2^
GO:BP	Nuclear chromosome segregation	2.47	32	2.06 × 10^−4^
GO:BP	Nuclear division	2.71	42	6.47 × 10^−7^
GO:BP	Nucleic acid biosynthetic process	1.75	131	2.42 × 10^−8^
GO:BP	Nucleic acid metabolic process	2.02	241	3.92 × 10^−25^
GO:BP	Nucleobase-containing compound biosynthetic process	1.80	168	6.72 × 10^−12^
GO:BP	Nucleobase-containing compound catabolic process	1.84	32	2.83 × 10^−2^
GO:BP	Nucleobase-containing compound metabolic process	1.99	308	3.62 × 10^−32^
GO:BP	Nucleobase-containing small molecule metabolic process	1.91	67	2.86 × 10^−5^
GO:BP	Nucleoside diphosphate metabolic process	2.91	14	2.17 × 10^−2^
GO:BP	Nucleoside monophosphate biosynthetic process	3.25	12	2.30 × 10^−2^
GO:BP	Nucleoside monophosphate metabolic process	2.90	12	4.90 × 10^−2^
GO:BP	Nucleoside phosphate biosynthetic process	2.09	35	2.32 × 10^−3^
GO:BP	Nucleoside phosphate metabolic process	1.96	61	3.63 × 10^−5^
GO:BP	Nucleoside triphosphate biosynthetic process	4.35	18	2.11 × 10^−5^
GO:BP	Nucleoside triphosphate metabolic process	3.36	31	5.03 × 10^−7^
GO:BP	Nucleotide biosynthetic process	2.05	34	3.69 × 10^−3^
GO:BP	Nucleotide metabolic process	2.00	59	3.40 × 10^−5^
GO:BP	Organelle assembly	1.63	79	8.36 × 10^−4^
GO:BP	Organelle fission	2.44	42	1.11 × 10^−5^
GO:BP	Organelle organization	1.38	265	8.36 × 10^−7^
GO:BP	Organic acid metabolic process	1.70	80	1.79 × 10^−4^
GO:BP	Organonitrogen compound biosynthetic process	2.54	213	1.45 × 10^−35^
GO:BP	Organonitrogen compound metabolic process	1.46	436	4.46 × 10^−17^
GO:BP	Oxidative phosphorylation	6.34	30	2.57 × 10^−14^
GO:BP	Oxoacid metabolic process	1.75	79	6.92 × 10^−5^
GO:BP	Peptidyl-amino acid modification	1.78	34	3.42 × 10^−2^
GO:BP	Peptidyl-proline modification	4.51	9	1.58 × 10^−2^
GO:BP	Positive regulation of apoptotic process	2.00	25	3.42 × 10^−2^
GO:BP	Positive regulation of signal transduction by p53 class mediator	9.66	5	2.30 × 10^−2^
GO:BP	Primary metabolic process	1.45	670	5.01 × 10^−32^
GO:BP	Protein metabolic process	1.41	327	6.60 × 10^−10^
GO:BP	Protein peptidyl-prolyl isomerization	6.37	8	3.90 × 10^−3^
GO:BP	Protein–DNA complex assembly	3.28	23	6.53 × 10^−5^
GO:BP	Protein–DNA complex organization	2.23	62	3.95 × 10^−7^
GO:BP	Protein–RNA complex assembly	4.26	40	1.03 × 10^−12^
GO:BP	Protein–RNA complex organization	4.20	41	8.95 × 10^−13^
GO:BP	Protein-containing complex assembly	2.07	106	1.19 × 10^−10^
GO:BP	Protein-containing complex organization	1.97	153	5.98 × 10^−14^
GO:BP	Proteinogenic amino acid biosynthetic process	3.09	13	2.14 × 10^−2^
GO:BP	Proteinogenic amino acid metabolic process	2.46	26	1.73 × 10^−3^
GO:BP	Proton motive force-driven ATP synthesis	6.20	11	1.68 × 10^−4^
GO:BP	Purine nucleoside diphosphate catabolic process	3.46	11	2.38 × 10^−2^
GO:BP	Purine nucleoside diphosphate metabolic process	3.61	12	1.02 × 10^−2^
GO:BP	Purine nucleoside triphosphate biosynthetic process	4.29	13	1.08 × 10^−3^
GO:BP	Purine nucleoside triphosphate metabolic process	3.25	25	2.63 × 10^−5^
GO:BP	Purine nucleotide biosynthetic process	1.95	26	3.69 × 10^−2^
GO:BP	Purine nucleotide metabolic process	1.90	48	1.11 × 10^−3^
GO:BP	Purine ribonucleoside diphosphate catabolic process	3.46	11	2.38 × 10^−2^
GO:BP	Purine ribonucleoside diphosphate metabolic process	3.38	11	2.75 × 10^−2^
GO:BP	Purine ribonucleoside triphosphate biosynthetic process	4.06	12	3.66 × 10^−3^
GO:BP	Purine ribonucleoside triphosphate metabolic process	3.28	24	3.71 × 10^−5^
GO:BP	Purine ribonucleotide biosynthetic process	2.09	23	3.25 × 10^−2^
GO:BP	Purine ribonucleotide metabolic process	2.03	41	1.00 × 10^−3^
GO:BP	Purine-containing compound biosynthetic process	1.93	27	3.50 × 10^−2^
GO:BP	Purine-containing compound metabolic process	1.86	50	1.20 × 10^−3^
GO:BP	Pyridine nucleotide catabolic process	3.46	11	2.38 × 10^−2^
GO:BP	Pyridine-containing compound catabolic process	3.38	11	2.75 × 10^−2^
GO:BP	Pyruvate metabolic process	2.87	14	2.38 × 10^−2^
GO:BP	rRNA metabolic process	2.48	38	2.68 × 10^−5^
GO:BP	rRNA processing	2.56	36	2.63 × 10^−5^
GO:BP	Recombinational repair	2.24	20	3.24 × 10^−2^
GO:BP	Regulation of DNA metabolic process	2.01	28	1.89 × 10^−2^
GO:BP	Regulation of DNA replication	3.74	13	3.86 × 10^−3^
GO:BP	Regulation of G2/M transition of mitotic cell cycle	3.44	15	2.78 × 10^−3^
GO:BP	Regulation of apoptotic process	1.53	60	2.68 × 10^−2^
GO:BP	Regulation of apoptotic signaling pathway	2.11	25	1.93 × 10^−2^
GO:BP	Regulation of cell cycle	2.05	78	2.06 × 10^−7^
GO:BP	Regulation of cell cycle G2/M phase transition	3.18	16	3.60 × 10^−3^
GO:BP	Regulation of cell cycle phase transition	2.32	39	8.76 × 10^−5^
GO:BP	Regulation of cell cycle process	2.13	58	5.99 × 10^−6^
GO:BP	Regulation of cellular response to stress	2.09	30	6.75 × 10^−3^
GO:BP	Regulation of chromosome organization	2.35	17	4.54 × 10^−2^
GO:BP	Regulation of chromosome segregation	3.10	14	1.27 × 10^−2^
GO:BP	Regulation of double-strand break repair	2.90	12	4.90 × 10^−2^
GO:BP	Regulation of metaphase/anaphase transition of cell cycle	3.76	10	2.42 × 10^−2^
GO:BP	Regulation of mitotic cell cycle	2.05	37	1.99 × 10^−3^
GO:BP	Regulation of mitotic cell cycle phase transition	2.28	26	5.00 × 10^−3^
GO:BP	Regulation of mitotic metaphase/anaphase transition	3.87	10	2.13 × 10^−2^
GO:BP	Regulation of mitotic sister chromatid separation	3.80	9	4.04 × 10^−2^
GO:BP	Regulation of translation	2.00	24	4.09 × 10^−2^
GO:BP	Respiratory electron transport chain	5.74	28	5.17 × 10^−12^
GO:BP	Response to stress	1.40	161	3.57 × 10^−4^
GO:BP	Ribonucleoprotein complex biogenesis	3.17	84	5.63 × 10^−19^
GO:BP	Ribonucleoside diphosphate catabolic process	3.38	11	2.75 × 10^−2^
GO:BP	Ribonucleoside diphosphate metabolic process	3.25	12	2.30 × 10^−2^
GO:BP	Ribonucleoside triphosphate biosynthetic process	4.03	14	1.00 × 10^−3^
GO:BP	Ribonucleoside triphosphate metabolic process	3.32	26	1.07 × 10^−5^
GO:BP	Ribonucleotide biosynthetic process	2.04	25	2.81 × 10^−2^
GO:BP	Ribonucleotide metabolic process	2.03	44	5.34 × 10^−4^
GO:BP	Ribose phosphate biosynthetic process	2.20	28	5.00 × 10^−3^
GO:BP	Ribose phosphate metabolic process	2.16	48	4.64 × 10^−5^
GO:BP	Ribosomal large subunit biogenesis	3.74	21	2.61 × 10^−5^
GO:BP	Ribosomal small subunit assembly	6.31	7	1.33 × 10^−2^
GO:BP	Ribosomal small subunit biogenesis	3.69	21	3.07 × 10^−5^
GO:BP	Ribosome assembly	5.71	19	9.60 × 10^−8^
GO:BP	Ribosome biogenesis	2.90	58	5.23 × 10^−11^
GO:BP	Sexual reproduction	1.74	51	4.56 × 10^−3^
GO:BP	Signal transduction in response to DNA damage	2.54	15	4.38 × 10^−2^
GO:BP	Sister chromatid segregation	2.91	20	1.62 × 10^−3^
GO:BP	Small molecule metabolic process	1.54	147	7.02 × 10^−6^
GO:BP	Spindle organization	2.62	32	6.68 × 10^−5^
GO:BP	tRNA aminoacylation	5.04	22	5.27 × 10^−8^
GO:BP	tRNA aminoacylation for protein translation	4.92	20	5.03 × 10^−7^
GO:BP	tRNA metabolic process	2.28	34	5.83 × 10^−4^
GO:BP	Tetrahydrofolate metabolic process	6.24	6	3.75 × 10^−2^
GO:BP	Translation	5.77	136	3.59 × 10^−65^
GO:BP	Translational elongation	4.77	12	8.57 × 10^−4^
GO:BP	Translational initiation	5.34	15	1.59 × 10^−5^
GO:BP	Tricarboxylic acid cycle	5.34	15	1.59 × 10^−5^
Cellular Component				
GO:CC	Arp2/3 protein complex	6.92	6	1.02 × 10^−2^
GO:CC	Sm-like protein family complex	3.21	16	1.16 × 10^−3^
GO:CC	U1 snRNP	5.10	7	1.67 × 10^−2^
GO:CC	U12-type spliceosomal complex	4.62	7	2.88 × 10^−2^
GO:CC	U2 snRNP	5.54	8	3.72 × 10^−3^
GO:CC	U2-type spliceosomal complex	3.64	15	5.33 × 10^−4^
GO:CC	U4 snRNP	10.39	6	1.12 × 10^−3^
GO:CC	U5 snRNP	7.55	6	6.68 × 10^−3^
GO:CC	Aminoacyl-tRNA synthetase multienzyme complex	8.08	7	1.12 × 10^−3^
GO:CC	Catalytic complex	1.64	188	1.31 × 10^−10^
GO:CC	Catalytic step 2 spliceosome	3.02	17	1.37 × 10^−3^
GO:CC	Centrosome	1.78	55	5.33 × 10^−4^
GO:CC	Chromatin	1.69	58	1.18 × 10^−3^
GO:CC	Chromosomal region	2.85	41	6.00 × 10^−8^
GO:CC	Chromosome	2.09	131	2.56 × 10^−14^
GO:CC	Chromosome, centromeric region	2.94	34	7.34 × 10^−7^
GO:CC	Cleavage furrow	4.82	8	9.12 × 10^−3^
GO:CC	Condensed chromosome	2.77	36	1.29 × 10^−6^
GO:CC	Condensed chromosome, centromeric region	2.70	24	3.03 × 10^−4^
GO:CC	Cytochrome complex	5.86	11	1.01 × 10^−4^
GO:CC	Cytoplasm	1.28	929	7.97 × 10^−28^
GO:CC	Cytosol	1.95	302	3.27 × 10^−30^
GO:CC	Cytosolic large ribosomal subunit	8.46	44	4.02 × 10^−29^
GO:CC	Cytosolic ribosome	8.63	76	4.32 × 10^−52^
GO:CC	Cytosolic small ribosomal subunit	9.54	31	1.85 × 10^−22^
GO:CC	Eukaryotic 43S preinitiation complex	9.23	8	9.12 × 10^−5^
GO:CC	Eukaryotic 48S preinitiation complex	11.08	8	1.63 × 10^−5^
GO:CC	Eukaryotic translation initiation factor 3 complex	8.90	9	2.36 × 10^−5^
GO:CC	Eukaryotic translation initiation factor 3 complex, eIF3m	11.08	4	3.24 × 10^−2^
GO:CC	Inner mitochondrial membrane protein complex	3.86	34	4.75 × 10^−10^
GO:CC	Intracellular anatomical structure	1.23	1265	8.08 × 10^−52^
GO:CC	Intracellular membrane-bounded organelle	1.28	905	4.52 × 10^−26^
GO:CC	Intracellular non-membrane-bounded organelle	1.72	459	4.71 × 10^−35^
GO:CC	Intracellular organelle	1.29	1087	1.05 × 10^−42^
GO:CC	Intracellular organelle lumen	1.94	275	3.97 × 10^−27^
GO:CC	Intracellular protein-containing complex	1.44	89	4.68 × 10^−3^
GO:CC	Kinetochore	2.89	24	1.01 × 10^−4^
GO:CC	Large ribosomal subunit	6.31	51	3.76 × 10^−26^
GO:CC	Membrane-bounded organelle	1.26	933	9.54 × 10^−25^
GO:CC	Membrane-enclosed lumen	1.94	275	3.97 × 10^−27^
GO:CC	Microtubule cytoskeleton	1.48	111	4.41 × 10^−4^
GO:CC	Microtubule organizing center	1.54	60	1.02 × 10^−2^
GO:CC	Mitochondrial envelope	2.13	73	3.18 × 10^−8^
GO:CC	Mitochondrial inner membrane	2.94	60	2.55 × 10^−12^
GO:CC	Mitochondrial matrix	3.48	50	4.42 × 10^−13^
GO:CC	Mitochondrial membrane	2.17	69	4.32 × 10^−8^
GO:CC	Mitochondrial protein-containing complex	3.58	59	2.73 × 10^−16^
GO:CC	Mitochondrial proton-transporting ATP synthase complex	6.43	13	4.00 × 10^−6^
GO:CC	Mitochondrial proton-transporting ATP synthase complex, coupling factor F(o)	5.93	6	2.21 × 10^−2^
GO:CC	Mitochondrial respirasome	5.66	9	1.12 × 10^−3^
GO:CC	Mitochondrial ribosome	3.68	21	9.69 × 10^−6^
GO:CC	Mitochondrial small ribosomal subunit	6.65	12	8.40 × 10^−6^
GO:CC	Mitochondrion	2.39	235	6.18 × 10^−36^
GO:CC	Mitotic spindle	2.16	17	4.58 × 10^−2^
GO:CC	Non-membrane-bounded organelle	1.72	460	2.54 × 10^−35^
GO:CC	Nuclear chromosome	1.95	22	3.89 × 10^−2^
GO:CC	Nuclear lumen	1.85	224	6.28 × 10^−19^
GO:CC	Nuclear protein-containing complex	1.60	136	7.62 × 10^−7^
GO:CC	Nucleolus	2.34	76	1.62 × 10^−10^
GO:CC	Nucleoplasm	1.80	169	1.02 × 10^−12^
GO:CC	Nucleus	1.36	561	2.14 × 10^−17^
GO:CC	Organellar ribosome	3.68	21	9.69 × 10^−6^
GO:CC	Organellar small ribosomal subunit	6.65	12	8.40 × 10^−6^
GO:CC	Organelle	1.26	1100	9.05 × 10^−39^
GO:CC	Organelle envelope	1.93	104	2.99 × 10^−9^
GO:CC	Organelle inner membrane	2.73	64	1.16 × 10^−11^
GO:CC	Organelle lumen	1.94	275	3.97 × 10^−27^
GO:CC	Oxidoreductase complex	4.24	19	4.39 × 10^−6^
GO:CC	pICln-Sm protein complex	9.89	5	8.41 × 10^−3^
GO:CC	Preribosome	2.74	20	1.16 × 10^−3^
GO:CC	Proteasome core complex	6.23	9	5.43 × 10^−4^
GO:CC	Protein–DNA complex	1.72	65	3.03 × 10^−4^
GO:CC	Protein-containing complex	1.54	570	5.76 × 10^−32^
GO:CC	Proton-transporting ATP synthase complex	6.21	13	5.94 × 10^−6^
GO:CC	Proton-transporting ATP synthase complex, coupling factor F(o)	5.19	6	3.99 × 10^−2^
GO:CC	Proton-transporting two-sector ATPase complex	3.58	15	6.27 × 10^−4^
GO:CC	Replication fork	3.53	13	2.44 × 10^−3^
GO:CC	Respirasome	5.25	11	3.03 × 10^−4^
GO:CC	Respiratory chain complex	5.86	11	1.01 × 10^−4^
GO:CC	Respiratory chain complex IV	5.70	7	9.06 × 10^−3^
GO:CC	Ribonucleoprotein complex	3.78	177	5.05 × 10^−54^
GO:CC	Ribosomal subunit	7.04	93	1.92 × 10^−53^
GO:CC	Ribosome	6.68	108	4.42 × 10^−59^
GO:CC	Small nuclear ribonucleoprotein complex	3.64	15	5.33 × 10^−4^
GO:CC	Small ribosomal subunit	8.11	41	3.74 × 10^−26^
GO:CC	Small-subunit processome	2.77	12	3.26 × 10^−2^
GO:CC	Spindle	2.33	45	4.67 × 10^−6^
GO:CC	Spliceosomal complex	2.45	37	1.52 × 10^−5^
GO:CC	Spliceosomal snRNP complex	3.59	14	1.12 × 10^−3^
GO:CC	Spliceosomal tri-snRNP complex	4.47	10	2.72 × 10^−3^
GO:CC	Translation preinitiation complex	9.59	9	1.15 × 10^−5^
GO:CC	Tricarboxylic acid cycle heteromeric enzyme complex	6.92	5	3.76 × 10^−2^
Molecular Function				
GO:MF	ATP-dependent activity, acting on DNA	2.32	23	1.60 × 10^−2^
GO:MF	NAD binding	3.78	17	5.51 × 10^−4^
GO:MF	NAD+ binding	6.22	7	2.33 × 10^−2^
GO:MF	RNA binding	2.37	182	6.82 × 10^−26^
GO:MF	Aminoacyl-tRNA ligase activity	5.08	20	5.57 × 10^−7^
GO:MF	Binding	1.05	999	2.45 × 10^−2^
GO:MF	Catalytic activity	1.13	553	9.04 × 10^−3^
GO:MF	Catalytic activity, acting on DNA	2.07	38	2.37 × 10^−3^
GO:MF	Catalytic activity, acting on RNA	1.89	51	1.40 × 10^−3^
GO:MF	Catalytic activity, acting on a nucleic acid	1.91	87	7.53 × 10^−7^
GO:MF	Catalytic activity, acting on a tRNA	3.17	29	8.40 × 10^−6^
GO:MF	Electron transfer activity	5.21	11	1.73 × 10^−3^
GO:MF	Heterocyclic compound binding	1.24	247	8.80 × 10^−3^
GO:MF	Identical protein binding	1.77	67	5.59 × 10^−4^
GO:MF	Isomerase activity	2.20	28	9.04 × 10^−3^
GO:MF	Ligase activity	2.63	45	6.96 × 10^−7^
GO:MF	Ligase activity, forming carbon-oxygen bonds	5.08	20	5.57 × 10^−7^
GO:MF	mRNA binding	1.99	42	2.32 × 10^−3^
GO:MF	Nucleic acid binding	1.49	340	2.94 × 10^−13^
GO:MF	Nucleoside phosphate binding	1.25	236	9.04 × 10^−3^
GO:MF	Nucleotide binding	1.25	236	9.04 × 10^−3^
GO:MF	Organic cyclic compound binding	1.30	544	9.27 × 10^−12^
GO:MF	Oxidoreductase activity	1.61	96	3.56 × 10^−4^
GO:MF	Oxidoreductase activity, acting on NAD(P)H	3.71	12	1.38 × 10^−2^
GO:MF	Oxidoreductase activity, acting on NAD(P)H, quinone or similar compound as acceptor	5.69	8	1.42 × 10^−2^
GO:MF	Oxidoreductase activity, acting on the CH-NH group of donors, NAD or NADP as acceptor	6.63	7	1.60 × 10^−2^
GO:MF	Proton transmembrane transporter activity	2.41	20	2.45 × 10^−2^
GO:MF	rRNA binding	6.56	24	3.02 × 10^−11^
GO:MF	Single-stranded DNA binding	2.84	17	1.33 × 10^−2^
GO:MF	Structural constituent of ribosome	7.30	95	1.45 × 10^−55^
GO:MF	Structural molecule activity	2.26	125	1.73 × 10^−15^
GO:MF	Translation elongation factor activity	5.53	7	4.38 × 10^−2^
GO:MF	Translation factor activity, RNA binding	4.28	25	2.61 × 10^−7^
GO:MF	Translation initiation factor activity	4.37	16	1.74 × 10^−4^
GO:MF	Translation regulator activity	3.92	32	1.15 × 10^−8^
GO:MF	Translation regulator activity, nucleic acid binding	3.93	26	5.88 × 10^−7^

**Table 6:** Significantly enriched KEGG pathways from DEGs identified at 12 and 24 hpi (results from the DAVID online resource).

Time Point	Regulation	KEGG Term	DEG Count	Fold Enrichment	*p*-Value (Adjusted)
12 hpi	Down	Ribosome	80	6.68	3.16 × 10^−49^
12 hpi	Down	Oxidative phosphorylation	37	3.22	1.08 × 10^−8^
12 hpi	Down	DNA replication	18	6.01	1.09 × 10^−8^
12 hpi	Down	Ribosome biogenesis in eukaryotes	27	4.03	1.09 × 10^−8^
12 hpi	Down	Spliceosome	30	2.50	1.25 × 10^−4^
12 hpi	Down	Nucleocytoplasmic transport	22	2.29	1.00 × 10^−2^
12 hpi	Down	Base excision repair	13	3.10	1.13 × 10^−2^
12 hpi	Down	Mismatch repair	9	4.29	1.13 × 10^−2^
12 hpi	Down	Nucleotide excision repair	14	2.86	1.29 × 10^−2^
12 hpi	Up	Steroid biosynthesis	10	6.14	1.65 × 10^−3^
12 hpi	Up	Autophagy—animal	29	2.34	2.12 × 10^−3^
12 hpi	Up	Cell cycle	27	2.30	3.90 × 10^−3^
12 hpi	Up	Influenza A	22	2.13	4.74 × 10^−2^
24 hpi	Down	Ribosome	88	5.54	2.81 × 10^−49^
24 hpi	Down	Oxidative phosphorylation	50	3.28	2.71 × 10^−13^
24 hpi	Down	Carbon metabolism	39	2.98	1.08 × 10^−8^
24 hpi	Down	Aminoacyl-tRNA biosynthesis	22	3.78	1.10 × 10^−6^
24 hpi	Down	Biosynthesis of amino acids	24	3.02	2.50 × 10^−5^
24 hpi	Down	Citrate cycle (TCA cycle)	15	4.36	2.50 × 10^−5^
24 hpi	Down	DNA replication	15	3.78	1.93 × 10^−4^
24 hpi	Down	Spliceosome	33	2.08	1.09 × 10^−3^
24 hpi	Down	Metabolic pathways	225	1.22	3.04 × 10^−3^
24 hpi	Down	Cell cycle	36	1.89	3.04 × 10^−3^
24 hpi	Down	Propanoate metabolism	12	3.24	7.53 × 10^−3^
24 hpi	Down	Fatty acid degradation	14	2.86	7.77 × 10^−3^
24 hpi	Down	Glycolysis/Gluconeogenesis	17	2.42	1.19 × 10^−2^
24 hpi	Down	One carbon pool by folate	9	3.78	1.35 × 10^−2^
24 hpi	Down	Nucleotide excision repair	15	2.31	3.73 × 10^−2^
24 hpi	Down	Pyruvate metabolism	12	2.59	4.20 × 10^−2^
24 hpi	Up	Steroid biosynthesis	11	5.15	1.92 × 10^−3^
24 hpi	Up	Lysosome	29	2.24	3.94 × 10^−3^
24 hpi	Up	Terpenoid backbone biosynthesis	9	4.43	1.73 × 10^−2^
24 hpi	Up	Glycosaminoglycan biosynthesis—heparan sulfate/heparin	10	3.90	1.73 × 10^−2^
24 hpi	Up	Protein processing in endoplasmic reticulum	30	1.94	1.73 × 10^−2^
24 hpi	Up	Autophagy—animal	30	1.85	3.19 × 10^−2^

## Data Availability

The raw sequencing read data (FastQ), gene expression counts, and total DEGs identified at 12 and 24 hpi have been deposited at the NCBI Gene Expression Omnibus (http://www.ncbi.nlm.nih.gov/geo) (accessed 2 January 2025) under accession number GSE286211. All the code/scripts in the entire analysis pipeline are available on GitHub (https://github.com/Abraham-Quaye/host_rna_seq) (accessed 2 January 2025).

## Supplementary Materials

The following supporting information can be downloaded at: https://www.mdpi.com/article/10.3390/v17030299/s1, Figure S1:Gel Electrophoresis of RT-qPCR validation reactions; Table S1: primers for RT-aPCR validation of RNA-seq Data; Table S2: Significantly enriched KEGG pathways from DEGs identified at 12 and 24 hpi.

## Abbreviations

The following are abbreviations used in this manuscript:

DAVID	Database for Annotation, Visualization and Integrated Discovery
DEG	Differentially Expressed Gene
ER	Endoplasmic Reticulum
ERAD	Endoplasmic Reticulum-associated Degradation
FPKM	Fragments Per Kilobase of transcript per Million mapped reads
GCN	Genome Copy Number
GO	Gene Ontology
HE	Hemorrhagic Enteritis
IMS	Immunosuppression
KEGG	Kyoto Encyclopedia of Genes and Genomes
ORF	Open Reading Frame
RNA-seq	RNA sequencing
RT-qPCR	Reverse Transcriptase Quantitative Polymerase Chain Reaction
THEV	Turkey Hemorrhagic Enteritis Virus
UPR	Unfolded Protein Response
VAS	Virginia Avirulent Strain
hpi	Hours Post-infection
