# Supplementary material for: Turkey B Cell Transcriptome Profile During Turkey Hemorrhagic Enteritis Virus (THEV) Infection Highlights Upregulated Apoptosis and Breakdown Pathways That May Mediate Immunosuppression
Source: Viruses. 2025 Feb 21;17(3):299. doi: 10.3390/v17030299 (PMC11945517; doi:10.3390/v17030299)
Supplement: Supplementary file 1 [file viruses-17-00299-s001.zip › viruses-3456941-supplementary.pdf]

Supplementary Materials

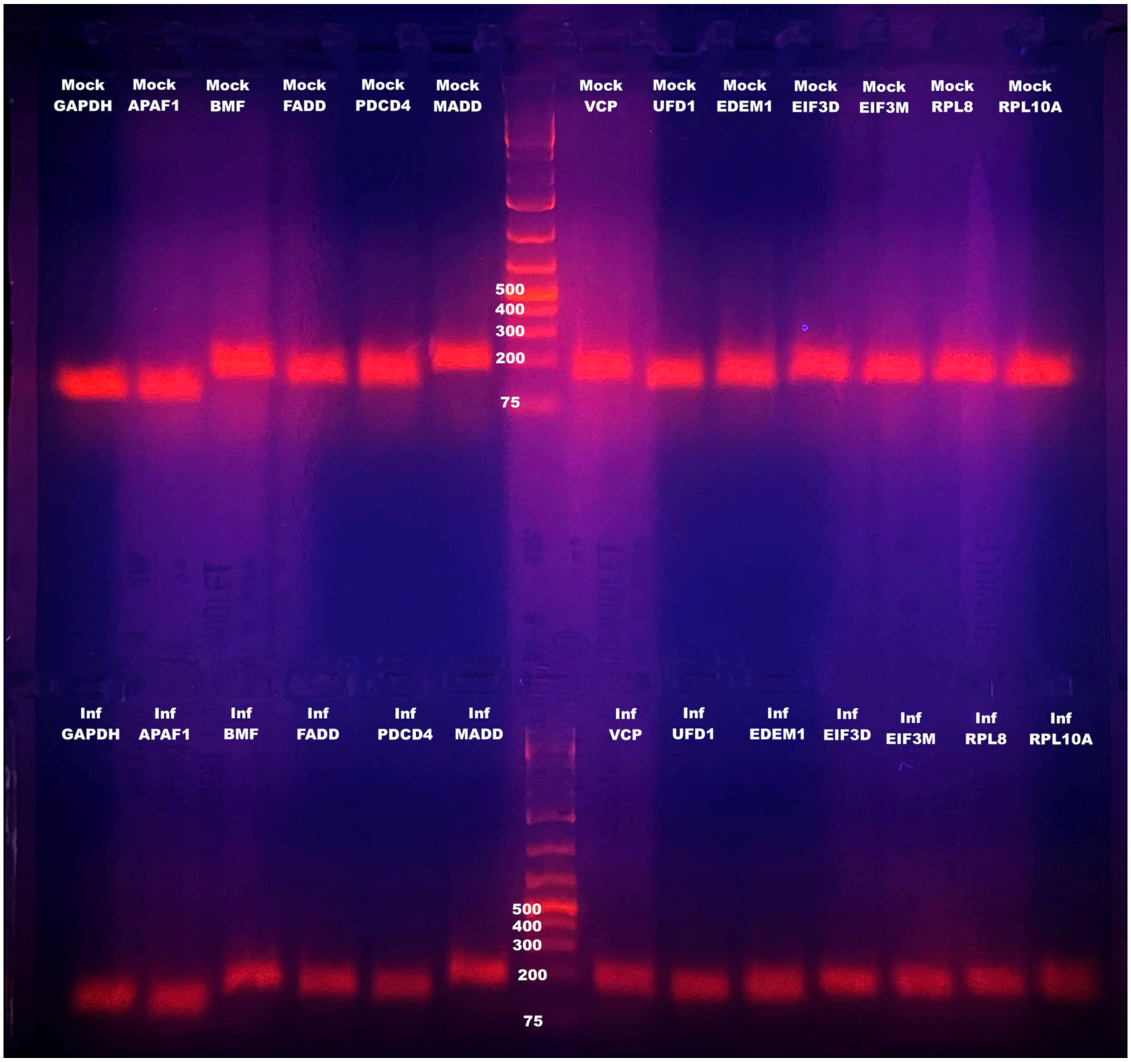

Figure S1: Gel Electrophoresis of RT-qPCR validation reactions. We run a gel electrophoresis of the RT-qPCR reactions in 2% agarose gel to confirm primer specificity. The 13 primer pairs all show excellent target specificity, each amplifying one amplicon of the expected size (see Table S1 for expected amplicon sizes of each primer pair). This was also confirmed in the RT-qPCR melt curves (not shown). Thermo Scientific™ generuler 1 kb plus DNA ladder was used. Mock-infected samples are shown in the top row prefixed with "mock" and infected samples are shown in the bottom row prefixed with "inf".

Table S1: Primers for RT-qPCR Validation of RNA-seq data

Entrez ID	Target Gene	Forward Primer	Reverse Primer	Amplicon Size
100549497	*APAF1*	GCTGCGCAAATACCCGAGGTC[ExJ]	GCCAGACACAGCATCTGTCACAC[ExJ]	133 bp

Entrez ID	Target Gene	Forward Primer	Reverse Primer	Amplicon Size
100550591	*BMF*	CGGAGACTCTTCTATGGGAATGCTGG[ExJ]	CTGCTGATGCCGCTGTATGTGG[ExJ]	189 bp
100543065	*EDEM1*	CTGGACTACAGGTGTTGATAGGAGACG[ExJ]	CCACTAACTCTGGCCTCAGTGG	159 bp
100545922	*EIF3D*	GCACAGAGGAACCTTCGGAGAG[ExJ]	GTCACGAGGCTTCTGCTGTGAC[ExJ]	180 bp
100545633	*EIF3M*	CTCTCAGACTGCAGCTACTGAGC[ExJ]	GTCTGTGCTGAGGTTCCAGTCAG	179 bp
100540536	*FADD*	GGAGCTCTGCAACTTCCTCATGG	CCTTCATGTCAGGCCACTCATCAG	167 bp
100303685	*GAPDH*[HK]	CACTATCTTCCAGGAGCGTGACC[ExJ]	CTGAGATGATAACACGCTTAGCACCAC	146 bp
100551463	*MADD*	GAGCTGACGAGGTTGAACTTGCTG[ExJ]	CTGGCTCCAATGATAACAAGGTAGTCG	200 bp
100547583	*PDCD4*	GCACAGTAGAAGTGGAGAATCTGAGTG[ExJ]	CTTCCTCAACCGCCTCTTTGC	161 bp
100544053	*RPL10A*	GGCACCGTCAGGCTGAAGTC[ExJ]	GGCATCGTACTTCTTAGCCAGCTTC[ExJ]	177 bp
100544011	*RPL8*	GCCGAGAGACATGGCTACATCAAGG	CAGCTGAGCTTTCTTGCCACAG[ExJ]	186 bp
104913522	*UFD1*	GTGGTCTGCTTCAACATCTGTGGTC[ExJ]	GATCTATGAGCTTCGGGTAATGGAGAC[ExJ]	154 bp
100548376	*VCP*	CAAGGCCATAGGAGTGAAGCCTC[ExJ]	CTCAGGTTGCTCTCAGACTCACC	171 bp

[HK]Control (house-keeping) gene[ExJ]Primer spans exon-exon junction;

Gene symbols: glyceraldehyde-3-phosphate dehydrogenase (*GAPDH*); apoptotic peptidase activating factor 1 (*APAF1*); Bcl2 modifying factor (*BMF*); FAS-associated protein with death domain (*FADD*); programmed cell death 4 (*PDCD4*); MAP kinase activating death domain (*MADD*); valosin containing protein (*VCP/p97*); Ubiquitin Recognition Factor in ER Associated Degradation 1 (*UFD1*); ER degradation enhancing alpha-mannosidase like protein 1 (*EDEM1*); eukaryotic translation initiation factor 3 subunit D and M (*EIF3D* and, *EIF3M*); ribosomal protein L8 and L10a (*RPL8*, and *RPL10A*).

Table S2: Significantly Enriched KEGG Pathways from DEGs identified at 12- and 24-hpi (Results from the gprofiler2 R package)

Time Point	Regulation	KEGG Term	DEG Count	P-value (Adjusted)
12-hpi	down	Ribosome	35	7.70e-24
12-hpi	down	DNA replication	11	5.07e-07
12-hpi	down	Oxidative phosphorylation	19	3.10e-04
12-hpi	down	Base excision repair	9	1.15e-03
12-hpi	down	One carbon pool by folate	6	1.27e-03
12-hpi	down	Mismatch repair	6	3.49e-03
12-hpi	down	Ribosome biogenesis in eukaryotes	9	1.77e-02
12-hpi	down	Nucleotide excision repair	8	3.36e-02
12-hpi	up	Autophagy - animal	13	2.09e-02
24-hpi	down	Ribosome	41	4.71e-28
24-hpi	down	Aminoacyl-tRNA biosynthesis	12	3.04e-04
24-hpi	down	Oxidative phosphorylation	22	4.35e-04
24-hpi	down	Base excision repair	9	1.15e-02
24-hpi	down	Carbon metabolism	14	3.14e-02
24-hpi	down	Propanoate metabolism	6	3.99e-02
24-hpi	up	Ubiquitin mediated proteolysis	17	7.26e-03
24-hpi	up	Steroid biosynthesis	5	2.63e-02
